# Trib1 deficiency causes brown adipose respiratory chain depletion and mitochondrial disorder

**DOI:** 10.1038/s41419-021-04389-x

**Published:** 2021-11-22

**Authors:** Xuelian Zhang, Bin Zhang, Chenyang Zhang, Guibo Sun, Xiaobo Sun

**Affiliations:** 1 Institute of Medicinal Plant Development, Peking Union Medical College and Chinese Academy of Medical Sciences, Beijing, 100193 China; 2 Key Laboratory of Bioactive Substances and Resources Utilization of Chinese Herbal Medicine, Ministry of Education, Beijing, 100193 China; 3 Beijing Key Laboratory of Innovative Drug Discovery of Traditional Chinese Medicine (Natural Medicine) and Translational Medicine, Beijing, 100193 China; 4 Key Laboratory of Efficacy Evaluation of Chinese Medicine Against Glyeolipid Metabolism Disorder Disease, State Administration of Traditional Chinese Medicine, Beijing, 100193 China

**Keywords:** Energy metabolism, Obesity

## Abstract

Tribbles homolog 1 (TRIB1) belongs to the Tribbles family of pseudokinases, which plays a key role in tumorigenesis and inflammation. Although genome-wide analysis shows that *TRIB1* expression is highly correlated with blood lipid levels, the relationship between *TRIB1* and adipose tissue metabolism remains unclear. Accordingly, the aim of the present study was to explore the role of TRIB1 on mitochondrial function in the brown adipose tissue (BAT). *Trib1*-knockout mice were established using clustered regularly interspaced short palindromic repeats (CRISPR)/Cas9 technology. The metabolic function of the BAT was induced by a β3-adrenoceptor agonist and the energy metabolism function of mitochondria in the BAT of mice was evaluated. *Trib1*-knockout mice exhibited obesity and impaired BAT thermogenesis. In particular, *Trib1* knockout reduced the ability of the BAT to maintain body temperature, inhibited β3-adrenoceptor agonist-induced thermogenesis, and accelerated lipid accumulation in the liver and adipose tissues. In addition, *Trib1* knockout reduced mitochondrial respiratory chain complex III activity, produced an imbalance between mitochondrial fusion and fission, caused mitochondrial structural damage and dysfunction, and affected heat production and lipid metabolism in the BAT. Conversely, overexpression of *Trib1* in 3T3-L1 adipocytes increased the number of mitochondria and improved respiratory function. These findings support the role of *Trib1* in regulating the mitochondrial respiratory chain and mitochondrial dynamics by affecting mitochondrial function and thermogenesis in the BAT.

## Introduction

Obesity is a global health problem that increases the risk of non-communicable diseases such as cardiovascular disease, diabetes, and cancer [1]. Obesity occurs when energy intake exceeds energy consumption for a prolonged period of time. At present, obesity is mainly treated by surgery or drugs to reduce the body’s energy intake; however, these approaches have many associated side effects [2]. Increasing energy consumption has emerged as a promising strategy for treating obesity. Brown adipocytes can convert stored fat into heat energy through mitochondrial uncoupling protein 1 (UCP1), which targets brown adipose tissue (BAT) activity. The mitochondria are enriched in brown adipocytes, which regulate energy metabolism in response to the external environment and hormones in vivo, and are the centers of glucose and lipid metabolism [3]. Therefore, normal mitochondrial function is essential for maintaining BAT function and metabolic homeostasis.

Mitochondria are highly dynamic organelles that change under different physiological conditions. The maintenance of mitochondrial dynamics is closely related to mitochondrial processes, including mitochondrial fusion, fission, and autophagy [4]; thus, mitochondrial dynamics are regulated by proteins involved in mitochondrial fusion and fission. The main proteins involved in mitochondrial fission are dynamin-related protein 1 (DRP1) and fission 1 (FIS1) [5–7], whereas those involved in mitochondrial fusion are mitofusin 1 (MFN1), mitofusin 2 (MFN2), and optic atrophy 1 (OPA1). MFN1 and MFN2 control mitochondrial outer membrane fusion and regulate OPA1 mitochondrial inner membrane fusion [8–10]. In addition to the classical phosphatase and tensin homolog-induced kinase 1-Parkin pathway and GTPases that regulate mitotic fusion proteins and influence mitochondrial dynamics [11, 12], oxidative phosphorylation has been suggested to be closely related to mitochondrial fusion. The knockout of mitochondrial fusion proteins can suppress oxidative phosphorylation, [13, 14] and reduce the activities of respiratory chain complexes I, III, and IV [14, 15]. Interestingly, oxidative phosphorylation can also affect mitochondrial dynamics. Some studies have found that oxidative phosphorylation can specifically induce mitochondrial inner membrane fusion without affecting outer membrane fusion [16]. Oxidative phosphorylation can also adjust the proportions of the fusion-promoting form (long, approximately 100 kDa) and non-fusion-promoting form (short, approximately 85 kDa) of OPA1 by affecting the hydrolysis of OPA1 [17]. Similarly, the in vitro addition of inhibitors of respiratory chain complexes II, III, IV, or ATP synthase (complex V) could effectively block mitochondrial intimal fusion [16]. Oxidative phosphorylation and mitochondrial dynamics can interact to regulate mitochondrial function in response to different physiological states. The adipose tissues of humans with obesity and obese mouse models were found to exhibit an imbalance of mitochondrial dynamics, decreased levels of oxidative phosphorylation, and mitochondrial dysfunction [18].

Tribbles homolog 1 (TRIB1) is a member of the Tribbles family of pseudokinases. Previous studies have found that TRIB1 can affect the occurrence of acute leukemia through the mitogen-activated protein kinase/extracellular signal-regulated kinase pathway and CCAAT enhancer-binding protein alpha (C/EBPα) [19, 20]. TRIB1 can also participate in the degradation of C/EBPα by interacting with constitutive photomorphogenic 1, thereby affecting the polarization of M2 macrophages in the spleen, bone marrow, and other organs of mice [21]. In addition, a genome-wide association analysis showed that *TRIB1* was associated with triglyceride (TG), total cholesterol (TC), and low-density lipoprotein (LDL) [22], linking TRIB1 with lipid metabolism. Further studies have confirmed that *TRIB1* overexpression can reduce the risk of coronary artery disease by reducing TG levels and inhibiting chemotaxis and cardiovascular remodeling [23]. Moreover, *Trib1* knockout in mice was reported to accelerate lipid accumulation in the liver and promote fatty liver development [24]. Therefore, we aimed to determine whether TRIB1 has a regulatory effect on adipose tissue and whether it has a direct impact on fat development and function. We designed a single-guide RNA using clustered regularly interspaced short palindromic repeats (CRISPR)/Cas9 technology and obtained *Trib1*-knockout mice via high-throughput electroporation of fertilized eggs. Here, we demonstrate that *Trib1* is essential for adipose tissue thermogenesis. Our results suggest that *Trib1* affects mitochondrial function and ultimately BAT heat production through the mitochondrial respiratory chain and mitochondrial dynamics.

## Results

### *Trib1* expression in the BAT increases in response to β3-adrenoceptor agonist stimulation

**Fig. 1:**
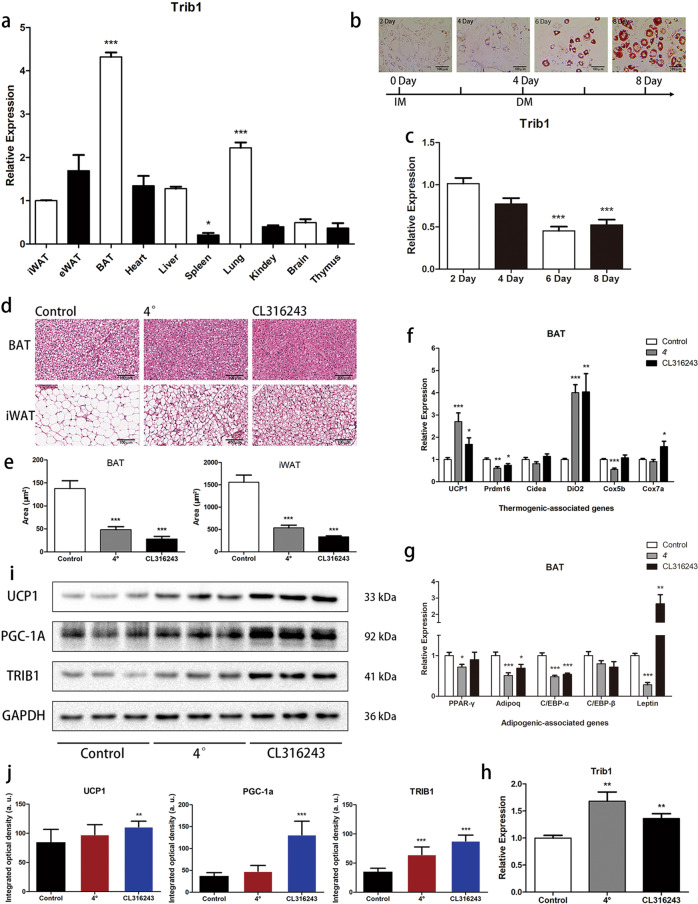
After adrenaline stimulation, the expression of *Trib1* in brown adipose increased. **a** The mRNA level of *Trib1* in a variety of organs and tissues of normal C57BL/6J mice. **b**, **c** Lipid drop oil red staining and mRNA level of *Trib1* in 3T3-L1 cells during adipogenesis. Scale bar: 100 μm. **d**, **e** H&E-stained and lipid droplet area statistics of BAT and iWAT sections obtained from the C57BL/6J mice treated with cold exposure or CL316243 (β3-adrenoceptor agonist). Scale bar: 100 μm. **f**–**h** After CL316243 treatment, the mRNA levels of *Trib1* and thermogenesis-related genes in BAT. **i**, **j** After CL316243 treatment, TRIB1 and thermogenic protein in BAT. BAT, brown adipose tissue; iWAT, inguinal white adipocyte tissue; eWAT, epididymal white adipocyte. In the bar figure, each data represents mean ± SEM (*n* = 6). One-way ANOVA multiple comparisons with Tukey’s test, ^*^P < 0.05, ^**^P < 0.01, ^***^P < 0.001 over control group.

Despite its well-known role in leukemia and M2 macrophages [21, 25], the physiological role of TRIB1 in the adipose tissue remains unclear. Therefore, we evaluated the distribution of *Trib1* in various tissues and organs of mice. Reverse transcription-quantitative polymerase chain reaction (RT-qPCR) analysis showed that *Trib1* was highly expressed in the BAT of mice (Fig. 1a and Supplemental Table 1). Moreover, with the formation of lipid droplets in 3T3-L1 mouse adipocytes (Fig. 1b), the expression level of *Trib1* decreased (Fig. 1c). The expression level of *Trib1* was also lower in the BAT of obese-hyperglycaemic *ob/ob* mice than in that of the control lean mice (Supplemental Fig. 1), suggesting that *Trib1* is closely related to the BAT.

The BAT is an important thermogenic tissue; therefore, we used a selective β3-adrenoceptor agonist (CL316243) to stimulate the thermogenic function of the BAT and explore the consequent changes in *Trib1* expression. C57BL/6 mice were intraperitoneally injected with CL316243 (1 mg/kg) every other day or cold exposed (room temperature at 4 °C) for 14 days. Hematoxylin and eosin staining showed that CL316243 and cold exposed induced the inguinal white adipose tissue from monocular lipid droplets to form multilocular lipid droplets and that the BAT lipid droplets became smaller, indicating increased lipolysis and thermogenesis (Fig. 1d, e). Moreover, the expression levels of genes and proteins related to thermogenesis were higher in CL316243-treated mice than those in the control group (Fig. 1f–j). With enhancement of the thermogenic function of the BAT, the expression level of *Trib1* further increased (Fig. 1h). Together, these findings suggested that *Trib1* may be involved in thermogenesis in the BAT.

### *Trib1* knockout prevents β3-adrenoceptor agonist-induced thermogenesis in the BAT and promotes mitochondrial structural damage

**Fig. 2:**
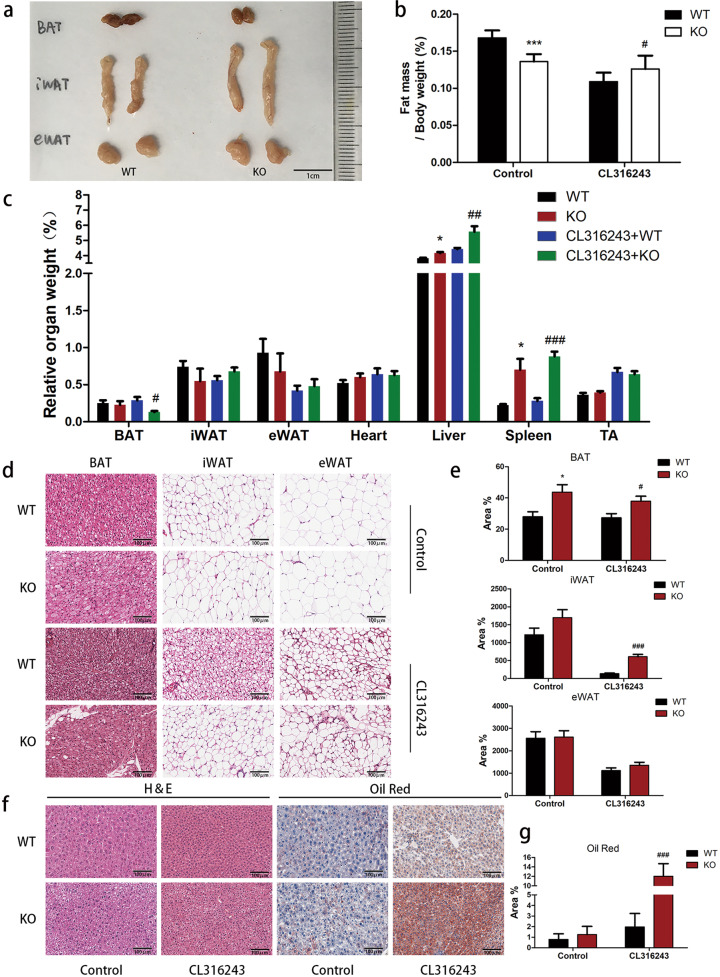
Decreased thermogenesis in *Trib1*-knockout mice. **a** Representative adipose tissue image of *Trib1*-knockout mice. Body composition (**b**) and ratio of tissue weight-to-bodyweight (**c**) of *Trib1* KO and WT mice treated with CL316243 or not. Representative hematoxylin and eosin (H&E) staining image (**d**) and adipocyte area (**e**) of BAT, iWAT, and eWAT from *Trib1* KO and WT mice treated with CL316243 or not. Scale bar: 100 μm. Representative hematoxylin and eosin (H&E) staining and oil red O staining image (**f**), and red staining area (**g**) of liver tissue from *Trib1* KO and WT mice treated with CL316243 or not. Scale bar: 100 μm. BAT, brown adipose tissue; iWAT, inguinal white adipocyte tissue; eWAT, epididymal white adipocyte. In the bar figure, each data represents mean ± SEM (*n* = 5). Indicated comparisons were made using Student’s paired *t*-test, ^*^P < 0.05, ^**^P < 0.01, ^***^P < 0.001 over *Trib1* WT mice; ^#^*P* < 0.05, ^##^*P* < 0.01, ^###^*P* < 0.001 over *Trib1* WT mice treated with CL316243.

To determine the effects of *Trib1* in the thermogenesis and metabolism of the BAT, we used CRISPR/Cas9 technology to obtain *Trib1*-knockout mice and identified their phenotypes (Supplemental Fig. 2). No significant difference in fat size was observed between wild-type and *Trib1*-knockout 8 weeks old mice fed normal food (Fig. 2a). However, after CL316243 treatment, the body fat mass weight to body weight ratio of *Trib1*-knockout mice increased significantly (Fig. 2b). The increased fat mass ratio in *Trib1*-knockout mice was closely related to the increase in inguinal white adipose tissue, epididymal white adipose tissue, and fat content in the liver (Fig. 2c). Further histological analysis showed that the adipocytes of *Trib1*-knockout mice were larger than those of wild-type mice after CL316243 treatment (Fig. 2d, e and Supplemental Fig. 3) and that more fat accumulated in the liver cells of *Trib1*-knockout mice than in those of wild-type mice (Fig. 2f, g). Compared with wild-type mice, fat accumulation in the hepatocytes of *Trib1*-knockout mice led to significant increases of aspartate aminotransferase, alkaline phosphatase, and alanine aminotransferase (Supplemental Fig. 4). CL316243 treatment can promote fat decomposition of the BAT [26]. However, the accumulation of large amounts of lipids in the adipose and liver indicates a disorder of lipid metabolism in the BAT.

**Fig. 3:**
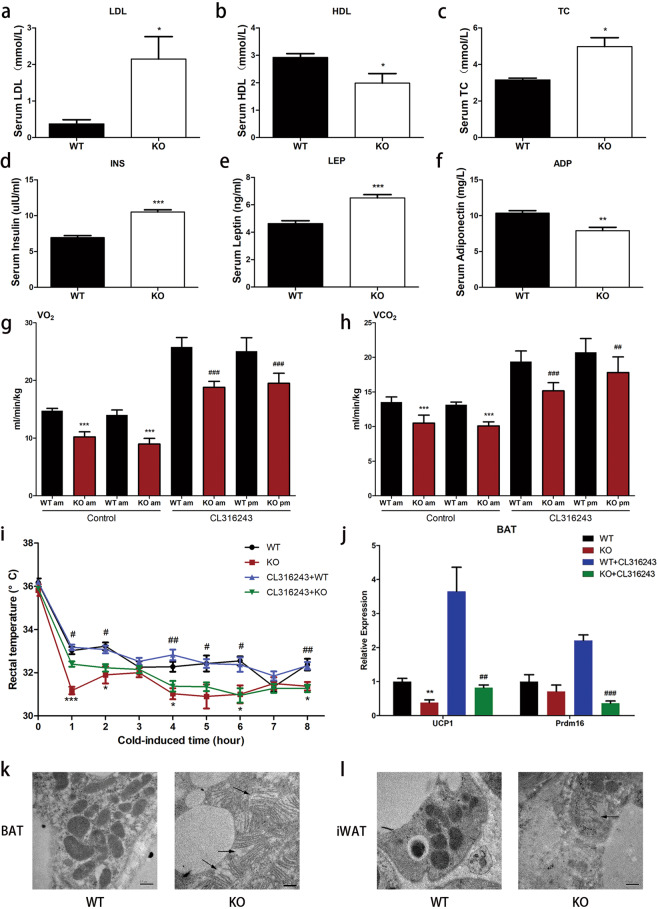
Mitochondrial structural damage of brown adipose in *Trib1*-knockout mice. Serum LDL (**a**), HDL (**b**), TC (**c**), insulin (**d**), leptin (**e**), and adiponectin (**f**) levels in *Trib1*-wild-type and knockout mice. Energy expenditure was evaluated by measurement of oxygen consumption (**g**) and carbon dioxide release (**h**) in *Trib1* KO and WT mice treated with CL316243 or not. After 8 h of cold exposure, the rectal temperature (**i**) of *Trib1* KO and WT mice treated with CL316243 or not. **j** The mRNA level of thermogenesis-related genes in BAT from *Trib1* KO and WT mice treated with CL316243 or not. TEM images of BAT (**k**) and iWAT (**l**) from *Trib1*-knockout mice treated with CL316243 or not. Black arrows indicated abnormal mitochondria. Scale bar: 0.5 μm. LDL, low-density lipoprotein; HDL, high-density lipoprotein; TC, total cholesterol; UCP1, uncoupling protein 1; Prdm16, PR/SET domain 16; BAT, brown adipose tissue; iWAT, inguinal white adipocyte tissue. In the bar figure, each data represents mean ± SEM (*n* = 5). Indicated comparisons were made using Student’s paired *t*-test, ^*^P < 0.05, ^**^P < 0.01, ^***^P < 0.001 over *Trib1* WT mice; ^#^*P* < 0.05, ^##^*P* < 0.01, ^###^*P* < 0.001 over *Trib1* WT mice treated with CL316243.

Furthermore, we measured the serum levels of LDL, HDL, and TC. Compared with wild-type mice, the expression levels of TC and LDL in *Trib1*-knockout mice increased (Fig. 3a, c), whereas the expression level of HDL decreased (Fig. 3b). Similarly, an increase in blood lipid levels was also observed in CL316243-treated *Trib1*-knockout mice (Supplemental Fig. 5). In addition, *Trib1*-knockout mice showed higher levels of insulin and leptin (Fig. 3d, e), but lower levels of adiponectin (Fig. 3f). These results further supported a disorder of lipid metabolism in *Trib1*-knockout mice.

Our results summarized above suggested that the fat metabolism function of the BAT is decreased under *Trib1* knockout; however, the effect of *Trib1* knockout on the thermogenic function of the BAT is not clear. Therefore, we next investigated the heat production ability of the *Trib1*-knockout mice. Indirect calorimetry measurements showed that the metabolic capacity of *Trib1*-knockout mice was lower than that of wild-type mice (Fig. 3g, h). Although treatment with CL316243 could improve the metabolic capacity of mice, the respiratory metabolic capacity of *Trib1*-knockout mice was still lower than that of the wild-type control under CL316243 treatment (Fig. 3g, h). We also evaluated the BAT thermogenesis of mice by cold adaptation. The mice were exposed to a temperature of 4 °C for 8 h and their rectal temperature was continuously recorded every hour. The *Trib1*-knockout mice exhibited a lower rectal temperature than the wild-type mice, indicating that the ability of the *Trib1*-knockout mice to produce heat and maintain body temperature was not as effective as that of wild-type mice (Fig. 3i). RT-qPCR showed that the expression levels of key genes related to heat production were significantly lower in the BAT of *Trib1*-knockout mice than in that of wild-type mice (Fig. 3j and Supplemental Table 1).

Because mitochondria are critical for heat production and fatty acid β-oxidation [27], the thermal damage and fat accumulation in the livers of *Trib1*-knockout mice motivated us to further study the effects of *Trib1* knockout on the structure and function of the mitochondria. Transmission electron microscopy showed that the mitochondria in the BAT of the control group were complete and oval; however, *Trib1* knockout resulted in the swelling of mitochondria and destruction of the mitochondrial membrane in the BAT (Fig. 3k, l). These results suggested that *Trib1* knockout induces mitochondrial structural damage, thereby impairing BAT thermogenesis.

### *Trib1* knockout leads to the decrease and dysfunction of mitochondrial assembly proteins in mice

**Fig. 4:**
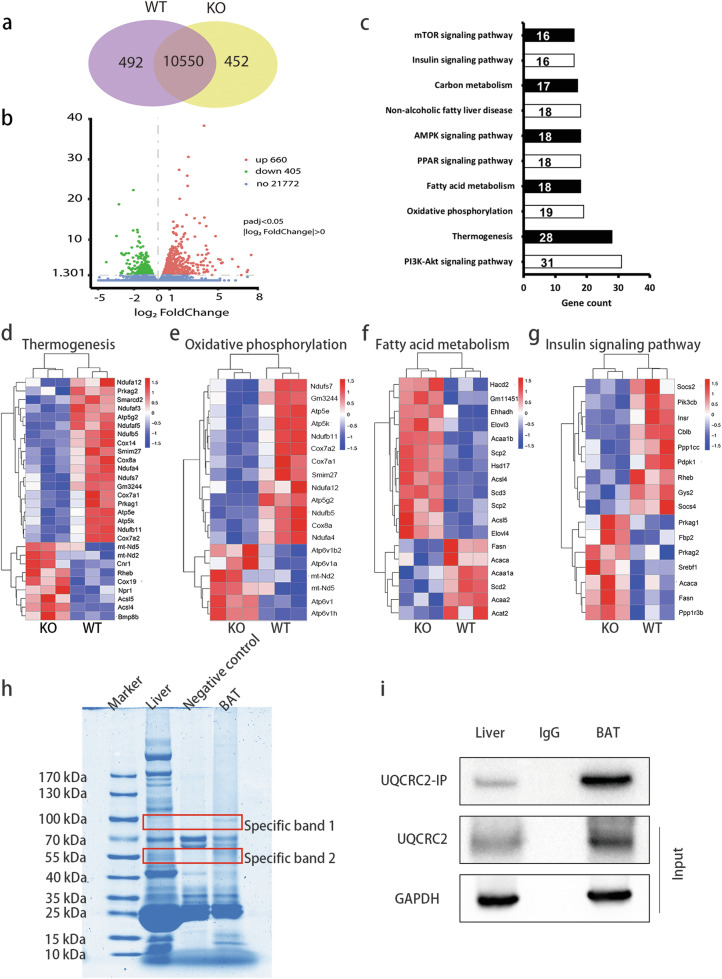
*Trib1* knockout leads to the decrease and dysfunction of mitochondrial assembly protein in mice. **a** The Venn diagram of RNA-seq indicated the number of differentially expressed genes between *Trib1*-knockout and wild-type mice, respectively (*n* = 3). **b** The volcano plot of RNA-seq showing up- and downregulated genes between *Trib1*-knockout and wild-type mice. **c** Kyoto Encyclopedia of Genes and Genomes analysis of downregulated pathway in *Trib1*-knockout mice and wild-type mice. Heat map of thermogenesis (**d**), oxidative phosphorylation (**e**), fatty acid metabolism (**f**), and insulin signaling pathway (**g**) gene clusters in *Trib1*-knockout and wild-type mice. Red and blue represent upregulation and downregulation expression, respectively. **h** The final elution (CoIP) samples were analyzed by Western blot and stained with Coomassie brilliant blue. The specific bands were used for mass spectrometry analysis. **i** The final elution (CoIP) samples were analyzed by Western blot and incubated with uqcrc2 primary antibody.

Mitochondria are important organelles involved in many biological functions, including glucose metabolism, lipid metabolism, and oxidative phosphorylation. We sequenced the genes in the BAT of wild-type and *Trib1*-knockout mice to explore the effects of *Trib1* knockout on mitochondrial function. A total of 11,042 and 11,002 genes showed greater than a two-fold expression level change in wild-type and *Trib1*-knockout mice, respectively (Fig. 4a); the volcano plot showed that 660 genes were upregulated and 405 genes were downregulated (Fig. 4b). In addition to the downregulated mitochondrial component genes such as those associated with the mitochondrial complex, mitochondrial inner membrane, and mitochondrial membrane protein complex (Supplemental Fig. 6), 19 downregulated genes were identified, including *Atp5e, Prkag2*, and *Cox8a*, which are related to the thermogenic signaling pathway. Moreover, genes related to oxidative phosphorylation, fatty acid metabolism, and insulin sensitivity were also downregulated (Fig. 4c–g). These results suggest that the mitochondrial structural damage induced by *Trib1* knockout leads to mitochondrial dysfunction, causing impaired thermogenesis and altered lipid metabolism. We also explored the TRIB1-glutathione-S-transferase fusion protein, demonstrating that TRIB1 can bind to UQCRC2, a mitochondrial respiratory chain complex subunit (Fig. 4h, i). The mitochondrial respiratory chain is involved in mitochondrial oxidative phosphorylation and in glucose and lipid metabolism [28]. As such, the mitochondrial dysfunction induced by *Trib1* knockout may be related to the mitochondrial respiratory chain.

### *Trib1* knockout decreases mitochondrial respiratory chain activity and disrupts mitochondrial dynamic homeostasis

**Fig. 5:**
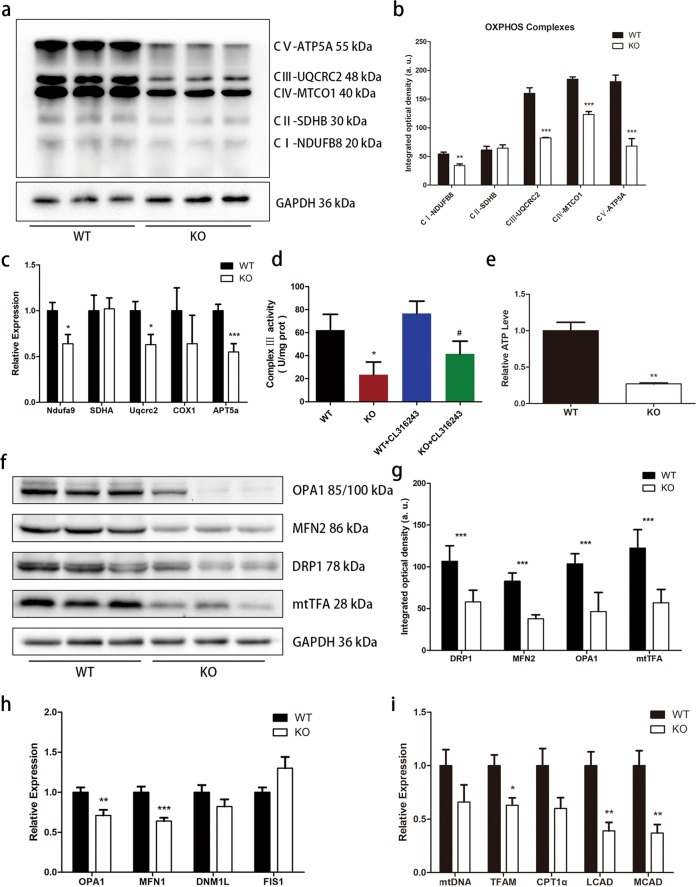
*Trib1* knockout results in decreased ATP production and disruption of mitochondrial homeostasis. **a**, **b** Western blots of mitochondrial electron transport chain complexes (complexes I–V) in brown adipose of *Trib1*-knockout and wild-type mice, and the results of optical density analyses. **c** The mRNA level of brown adipose respiratory chain complex in *Trib1*-knockout and wild-type mice. **d** Mitochondrial complex III activity of brown adipose in *Trib1* KO and WT mice treated with CL316243 or not. **e** ATP level of brown adipose in *Trib1*-knockout and wild-type mice. **f**, **g** Expression of mitochondrial fusion and fission proteins in brown adipose tissue of *Trib1*-knockout and wild-type mice, and the results of optical density analyses. **h** Mitochondrial fusion and fission gene mRNA level in *Trib1*-knockout and wild-type mice. **i** Mitochondrial copy number and fatty acid oxidation gene mRNA level of brown adipose in *Trib1*-knockout and wild-type mice. DRP1, dynamin-related protein 1; FIS1, fission protein 1; Mfn1, mitofusin 1; Mfn2, mitofusin 2; OPA1, optic atrophy 1; TFAM, transcription factor; CPT1α, carnitine palmitoyltransferase 1 A; MCAD, medium-chain acyl-CoA dehydrogenases; LCAD, long-chain acyl-CoA dehydrogenases. In the bar figure, each data represents mean ± SEM (*n* = 5). Indicated comparisons were made using Student’s paired *t*-test, ^*^P < 0.05, ^**^P < 0.01, ^***^P < 0.001 over *Trib1* WT mice; ^#^*P* < 0.05, ^##^*P* < 0.01, ^###^*P* < 0.001 over *Trib1* WT mice treated with CL316243.

UQCRC2 is a subunit of mitochondrial respiratory chain complex III [29]. Since we found that TRIB1 interacts with UQCRC2, we further explored the effects of *Trib1* on the mitochondrial respiratory chain. Western blot analysis and the RT-qPCR results showed that the expression levels of proteins and genes in mitochondrial respiratory chain complexes I, III, and V were lower in the BAT of *Trib1*-knockout mice than in those of wild-type mice (Fig. 5a–c). In addition, the activity of mitochondrial respiratory chain complex III was lower in the BAT of *Trib1*-knockout mice than in that of wild-type mice. After stimulation with CL316243, the activity of mitochondrial respiratory chain complex III increased slightly in *Trib1*-knockout mice, but was still lower than that in the wild-type control group (Fig. 5d).

Mitochondrial fusion is dependent on mitochondrial respiratory activity, and inhibition of respiratory chain complexes II, III, IV, or V can effectively prevent intimal fusion [16]. Therefore, we measured the ATP content in the BAT, showing that *Trib1* knockout significantly reduced ATP levels in the BAT (Fig. 5e). Subsequently, we measured the expression of OPA1, a key protein involved in mitochondrial intimal fusion and fission. The expression level of OPA1 decreased in the BAT of *Trib1*-knockout mice. In addition, the expression levels of mtTFA, MFN2, and DRP1 were lower in *Trib1*-knockout mice than in wild-type mice (Fig. 5f–h). These findings suggest that the decrease in mitochondrial respiratory chain activity affects mitochondrial dynamics and leads to mitochondrial structural damage. We further measured the expression of genes related to fatty acid β-oxidation. RT-qPCR showed that the expression levels of long-chain acetyl-CoA dehydrogenase and medium-chain acetyl-CoA dehydrogenase decreased in *Trib1*-knockout mice, indicating the inhibition of mitochondrial fatty acid β-oxidation (Fig. 5i and Supplemental Table 1). This result is consistent with previous observations in which knockout mice exhibited hyperlipidaemia and fat accumulation in the liver. Overall, these findings suggest that *Trib1* knockout disrupts mitochondrial dynamic homeostasis by affecting mitochondrial respiratory chain activity, leading to mitochondrial structural damage and lipid metabolism dysfunction.

### *Trib1* overexpression in 3T3-L1 adipocytes enhances respiratory metabolism

**Fig. 6:**
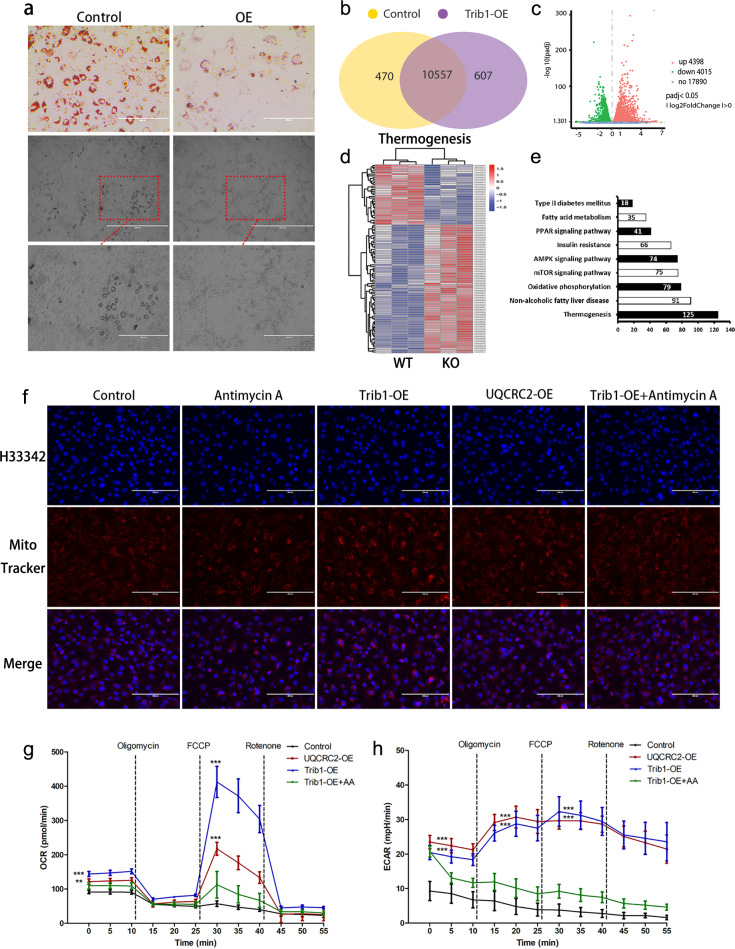
Overexpression of *Trib1* reduces lipid droplet accumulation in 3T3-L1 cells and increases respiratory metabolism. **a** After overexpression of *Trib1*, 3T3-L1 cells were stained with oil red staining and lipid droplets under natural light. Oil red scale bar: 400 μm; Lipid droplets under natural light scale bar: 1000 μm/400 μm. **b** The Venn diagram of RNA-seq indicated the number of differentially expressed genes between 3T3-L1 cells control group and *Trib1* overexpressing group, respectively (*n* = 3). **c** The volcano plot of RNA-seq showing up- and downregulated genes between 3T3-L1 cells control group and *Trib1* overexpressing group. **d** Heat map of thermogenesis gene clusters in 3T3-L1 cells control group and *Trib1* overexpressing group. Red and blue represent upregulation and downregulation expression, respectively. **e** Kyoto Encyclopedia of Genes and Genomes analysis of upregulated pathway in 3T3-L1 cells control group and *Trib1* overexpressing group. **f** Cells were stained with MitoTracker and Hoechst 33342. Scale bar: 200 μm. **g**, **h** Seahorse XF24 mitochondrial stress analyses for OCR in 3T3-L1 cells treated by overexpression of *Trib1* (*Trib1*-OE), Uqcrc2 (Uqcrc2-OE), and inhibitor (*Trib1*-OE + AA). OE, overexpression; AA, antimycin A. Each data represents mean ± SEM (*n* = 6). One-way ANOVA multiple comparisons with Tukey’s test, ^*^P < 0.05, ^**^P < 0.01, ^***^P < 0.01over control group.

The results summarized above demonstrated that *Trib1* knockout leads to mitochondrial dysfunction in the BAT of mice, causing impaired heat production and hyperlipidaemia. However, it is uncertain whether such dysfunction is due to a combination of *Trib1* knockout in other tissues such as the liver and muscle. Therefore, we overexpressed *Trib1* in 3T3-L1 preadipocytes and induced the differentiation and maturation of these cells (Supplemental Fig. 7). Oil Red O staining showed that the number of lipid droplets significantly reduced in the adipocytes with overexpressed *Trib1* (Fig. 6a and Supplemental Fig. 8a). However, there was no significant difference in lipid droplet differentiation between *Trib1*-knockdown and normal 3T3-L1 cells (Supplemental Fig. 8b). This result may be related to the low expression of *Trib1* during 3T3-L1 maturation (Fig. 1b). RNA sequencing revealed that the number of genes with a greater than two-fold expression change under the control and *Trib1* overexpression condition was 11,027 and 11,164, respectively (Fig. 6b); the volcano plot showed that 4398 genes were upregulated, whereas 4015 genes were downregulated (Fig. 6c). Genes related to heat production and oxidative phosphorylation were highly expressed in cells with overexpressed *Trib1* (Fig. 6d, e and Supplemental Table 2).

Using MitoTracker, we observed the mitochondrial changes after overexpressing *Trib1* and *Uqcrc2*, demonstrating that the number of mitochondria increased; however, this increase was suppressed by the addition of a mitochondrial respiratory chain complex III inhibitor (Fig. 6f and Supplemental Fig. 9). To determine the direct effect of *Trib1* overexpression on mitochondrial function, we evaluated oxidative phosphorylation and glycolysis ability by measuring oxygen consumption rate (OCR) and extracellular acidification rate (ECAR), respectively, in a seahorse assay. The OCR and ECAR in the *Trib1* overexpression group were significantly higher than those in the control group. Both basal and maximal OCR (after carbonyl cyanide-4-(trifluoromethoxy) phenylhydrazone treatment) in the *Trib1* overexpression group were higher than those in control group, but the function of *Trib1* was inhibited by the addition of antimycin A (Fig. 6g, h). These findings suggest that *Trib1* can enhance metabolic function by improving mitochondrial function, in line with the result showing that *Trib1* knockout impairs BAT thermogenesis. Overall, these findings suggest that *Trib1* can be an important target for regulating BAT function.

## Discussion

Our results showed that the *Trib1* knockout can damage adaptive thermogenesis and induce hyperlipidaemia and fatty liver in mice. Our results also revealed that *Trib1* overexpression in 3T3-L1 cells can reduce the insulin-induced accumulation of lipid droplets, increase the number of mitochondria, and improve respiratory metabolism in adipocytes, likely because *Trib1* knockout reduces mitochondrial respiratory chain activity, resulting in an imbalance in mitochondrial fusion and fission with subsequent mitochondrial structural damage and lipid metabolism dysfunction. Taken together, our results indicate that *Trib1* regulates the function of mitochondria and brown adipocytes in mice, suggesting its potential as a new target for the treatment of metabolic diseases such as hyperlipidaemia, fatty liver, and obesity.

Chambers et al. [30] performed a genome-wide association study in 61 089 individuals, identifying 42 loci associated with concentrations of liver enzymes in the plasma; among these, *TRIB1* was associated with glucose, carbohydrate, and lipid metabolism. Furthermore, Varbo et al. [31] genotyped >71,000 individuals, finding that the *TRIB1*-rs2954029 locus was associated with lipid levels and a risk of myocardial infarction in the general population. Moreover, liver-specific *Trib1*-knockout mice exhibited an increased plasma TG content and accelerated fatty liver development [24]. A recent study showed that berberine can reduce plasma TG levels in LDL receptor-deficient mice by upregulating the expression of liver *Trib1* [32]. These findings suggest that the expression of *Trib1* is negatively correlated with lipid levels in the serum and the liver. Our study revealed that *Trib1* knockout induced mitochondrial dysfunction in the BAT, accompanied by elevated blood lipid levels and fatty liver. These results provide new insight into the genetic mechanisms and pathways influencing lipid metabolism.

Interestingly, homozygous *Trib1*-knockout mice had a low survival rate (Supplemental Fig. 10). Previous studies have reported that *Trib1* haploinsufficiency protects against high-fat diet-induced obesity in mice, which was considered to be related to inhibition of the high-fat diet-mediated increase in proinflammatory gene expression due to *Trib1* knockout [33]. In contrast, we found no significant differences in body weight or body fat between wild-type and *Trib1*-haploinsufficient mice (Supplemental Fig. 11), although *Trib1*-knockout mice exhibited hyperlipidaemia and obesity even with a normal diet. We also found high expression of inflammatory cytokines in *Trib1*-knockout mice (Supplemental Fig. 12), which is consistent with a previous study showing that mice lacking *Trib1* in hematopoietic cells exhibited higher expression of proinflammatory cytokine genes [21].

Mitochondrial complexes can regulate mitochondrial function through reactive oxygen species production and oxidative phosphorylation [34, 35]. Mitochondrial fusion and fission can restore the function of damaged mitochondria. Inhibition of mitochondrial complexes can directly impair OPA1-regulated mitochondrial fusion [17]. Disruption of the fusion–fission balance leads to the accumulation of damaged mitochondria, causing mitochondrial functional defects [35, 36], including decreased oxidation of fatty acids, decreased expression of genes involved in oxidation, and decreased activity of the electron transport chain, contributing to the development of chronic metabolic diseases such as obesity and diabetes [37]. In our study, we found that *Trib1* has an effect on the mitochondrial respiratory chain complex III subunit UQCRC2 and that *Trib1* knockout reduces the enzymatic activity of mitochondrial respiratory chain complex III, leading to abnormal mitochondrial lipid metabolism and lipid accumulation in the liver. Similarly, Tomašić et al. [38] found that mutation of *BCS1L*, the gene encoding mitochondrial respiratory chain complex III, can induce the accumulation of liver fat. In addition, we found that overexpression of *Trib1* in 3T3-L1 adipocytes significantly enhanced respiratory metabolism, which was blunted in the presence of a mitochondrial respiratory chain complex III inhibitor. Some studies have found that UQCRC1 overexpression can accelerate the catalytic rate of complex III and improve mitochondrial respiratory capacity in cells [39]. Moreover, treatment of 3T3-L1 cells with a mitochondrial respiratory chain complex III inhibitor increased TG accumulation and decreased the ATP content and β oxidation level [40].

A chromatin immunoprecipitation assay showed that TRIB1 can bind to chromatin in 3T3-L1 preadipocytes, suggesting that TRIB1 is a potential direct transcription regulator [41]. However, the specific regulatory effects of TRIB1 on UQCRC2 requires further research. In addition, whether the specific overexpression of *Trib1* in the mouse BAT can enhance heat production by enhancing mitochondrial function and prevent obesity must be further investigated.

Overall, our results suggest that *Trib1* influences adipose tissue thermogenesis by regulating mitochondrial respiratory chain activity and mitochondrial dynamic homeostasis. Controlling the activity of TRIB1 through diet or pharmacotherapy can improve the thermogenic function of brown and beige fat as a new potential treatment strategy for obesity.

## Methods

### Animals and treatment

All animal experiments were performed in accordance with the guidelines of the National Institutes of Health Guide for the Care and Use of Laboratory Animals (NIH Publication No. 85-23, revised 1996) and were approved by the Experimental Laboratory Animal Committee of the Institute of Medicinal Plant Development, Peking Union Medical College.

CRISPR/Cas9-mediated *Trib1*-knockout mice were produced by Cyagen Biosciences Inc. (Suzhou, China). Cas9 and sgRNA were co-injected into fertilized eggs for knockout mouse production. The sgRNA target sequences included: sgRNA1 (5′-TTGCGCGAGGCTCGCGGCATGGG-3′) and sgRNA2 (5′-GCATAGGGTTTGGTAACCCGAGG-3′). Founder mice were screened for the presence of *Trib1* knockout by sequencing the PCR products amplified using the following primers: Trib1-sense (5′-GCTTGGGTTTGGCAGAGCAGATAAG-3′) and Trib1-antisense (5′-GTGCTAACTTCGGTATGTCCTCAGC-3′). *Trib1*-knockout mice were mated with wild-type C57BL/6 mice to obtain heterozygous *Trib1* mice. These mice were then mated for at least three generations to obtain *Trib1*-knockout and wild-type mice.

Male C57BL/6J mice (8 weeks old) were purchased from Shanghai Slac Laboratory Animal Co. Ltd. (Shanghai, China). The mice were maintained under standard laboratory conditions (room temperature at 22 °C, humidity of 60% with a 12 h light/dark cycle) and fed with a standard pellet diet and water *ad libitum*. After 1 week of adaptation, the mice were randomly divided into four groups (*n* = 6): (1) C57BL/6J group (control); (2) C57BL/6J + cold exposed group (4 °C); (3) C57BL/6J + CL316243 1 mg/kg group. C57BL/6 mice were intraperitoneally injected with CL316243 (1 mg/kg) every other day or cold exposed (room temperature at 4 °C) for 14 days.

### Cell culture

3T3-L1 preadipocytes were purchased from Peking Union Medical College (1101MOU-PUMC000155). 3T3-L1 fibroblasts were cultured in Dulbecco’s modified Eagle’s medium containing 10% fetal bovine serum. The cells were incubated at 37 °C in 5% CO_2_ and the medium was changed every 2 days. When the confluency rate reached 70–80%, the cells were passaged. 3T3-L1 cells began to differentiate after 2 days of growth and fusion. The induction medium contained 500 μM 3-isobutyl-1-methylxanthine, 0.25 mM dexamethasone, and 8 μg/ml insulin, and the cells were cultured for 4 days. The differentiation medium contained 0.1 nM triiodothyronine and 5 μg/ml insulin, and the cells were cultured for 4 days.

### Western blot analysis

Animal tissues and cells were lysed with cell lysis buffer containing 1% protease inhibitor and phosphatase inhibitor. The lysate was centrifuged at 12,000 rpm and 4 °C for 30 min to remove insoluble substances. The protein concentration was determined using the bicinchoninic acid quantitative method. The gel concentration was selected based on the molecular weight of the target protein. The sample size per hole was 20 μg, and the same amount of protein in each sample was separated via 12% sodium dodecyl sulfate-polyacrylamide gel electrophoresis and transferred to a nitrocellulose membrane. The membrane was blocked with 5% skim milk blocking buffer for 2 h at room temperature and incubated with the following primary antibodies overnight at 4 °C: UCP1 (Abcam, ab10983, 1:1000), PGC-1A (Abcam, ab54481, 1:1000), Trib1 (Santa Cruz Biotechnology, sc-393536, 1:200), UQCRC2 (Abcam, ab203832, 1:1000), OPA1 (Proteintech, 27733-1-AP, 1:1000), MFN2 (Proteintech, 12186-1-AP, 1:1000), DRP1 (Proteintech,12957-1-AP, 1:2000), mtTFA (Abcam, ab47517, 1:500), and GAPDH (Abcam, ab9485, 1:5000). The membrane was then washed three times with Tris-buffered saline with Tween 20, incubated with the secondary antibody at 1:2000 dilution for 2 h, and then washed with Tris-buffered saline with Tween 20. Finally, the imprints were observed via enhanced chemiluminescence using a Bio-Rad imaging system (Bio-Rad, Hercules, CA, USA).

### RNA extraction and RT-qPCR

Trizol (1 ml) was added to the animal tissues and cells. The tissues were ground evenly, 0.2 ml chloroform was added, shaken vigorously for 15 s, allowed to stand at room temperature for 5 min, and centrifuged at 12,000 rpm and 4 °C for 15 min. The aqueous layer was added with an equal volume of isopropanol, mixed upside down, allowed to stand at room temperature for 10 min, and then centrifuged at 12,000 rpm and 4 °C for 10 min. The supernatant was washed with 75% alcohol and centrifuged. The alcohol solution was discarded, and an appropriate amount of enzyme water was added to precipitate the RNA. The nucleic acid concentration was then determined. A reverse transcription kit (RR036A; Takara Bio, Shiga, Japan) was used for reverse transcription, and a fluorescence quantitative kit (RR820A; Takara Bio) was used for fluorescence quantitative analysis.

### RNA sequencing

Total RNA was extracted from the BAT of 8 weeks old wild-type and knockout mice for RNA sequencing. Sequencing was performed by Tianjin Novogene Bioinformatics Technology Co., Ltd. (Tianjin, China). First, a common transcriptome library was constructed. Then, 150-bp double-end sequencing was performed using an Illumina HiSeq platform (Illumina, San Diego, CA, USA). Finally, differential expression analysis, principal component analysis, Kyoto Encyclopedia of Genes and Genomes enrichment analysis, and protein interaction network analysis were performed. The DESeq R package (1.10.1) was used for differential expression analysis; the significance threshold was set at P < 0.05. Heat maps were produced using online tools (http://heatmapper.ca/).

### Histology and immunostaining

The tissues were fixed in paraformaldehyde for 2 days. After the tissues were rinsed and dehydrated, the paraffin-embedded sections were dried at 60 °C for 1 h, dewaxed with xylene, hydrated with different gradients of ethanol, and washed with water. For hematoxylin and eosin staining, the sections were soaked in hematoxylin dye for 5–20 min to stain the nuclei, washed with water, a weak alkaline aqueous solution was added for 30–60 s, and then the sections were rinsed with water for 5–10 min. Finally, the fully hydrated sections were stained with eosin for 15 min to stain the cytoplasm, eluted with gradient ethanol, made transparent with xylene, and then sealed. For immunohistochemical analysis, sections treated with blocking solution for 20 min at room temperature to block non-specific binding were incubated with antibodies against UCP1 (1:100) for 60 min at room temperature. Sections washed in phosphate-buffered saline (PBS) sections were incubated for 30 min at room temperature with horseradish peroxidase–conjugated antibodies against rabbit IgG. The color reaction was developed using 3,3′-diaminobenzidine substrate–chromogen solution, and the sections were lightly counterstained with Mayer hematoxylin before dehydration and mounting.

### Oil Red O staining of adipocytes

After washing the cells with PBS once, the cells were fixed with 4% paraformaldehyde at 4 °C for more than 30 min. The Oil Red O stock solution was diluted with water (3:2) and filtered. The cells were washed with PBS three times, stained with the Oil Red O working solution, and incubated for 30 min. After washing with PBS three times, the cells were observed under a microscope.

### Indirect calorimetry and energy expenditure calculation

To measure the basal oxygen consumption (*V*O_2_), carbon dioxide production (*V*CO_2_), and respiratory exchange rate (RER) in mice, a gas analyzer (LE 405 Gas Analyzer; Panlab, Cornellà de Llobregat, Spain) and Metabolism software (Panlab) were used. The mice were placed in metabolic cages (Oxylet Pro; Panlab) for 24 h, and *V*O_2_ and energy expenditure were measured after 25 h. The mice were maintained under a 12 h light/dark cycle with lights on at 6 AM and off at 6 PM and were allowed free access to food and water.

### Co-immunoprecipitation

The *Trib1* plasmid with a GST tag was transferred into *Escherichia coli*, and the culture broth was collected after culture expansion. The bacterial cells were subjected to ultrasound and centrifugation, and the supernatant of the fusion protein was collected. The collected protein supernatant was added to the prepared GST agarose gel at 40–50 drops/min to fully integrate the protein supernatant. The gel was washed, added to the extracted BAT protein, and incubated at a low temperature overnight. After centrifugation at a low speed, the gel was washed with buffer five to six times. After removing the upper clear liquid from the agarose gel, an appropriate amount of loading buffer and boiling water were added for 5 min. Western blot analysis was then performed.

### Evaluation of oxidative phosphorylation in cultured adipocytes

After transfection of the *Trib1/Uqcrc2* plasmid into 3T3-L1 cells, the cells were induced to differentiate and mature. After 8 days, the mature adipocytes were digested, counted, and seeded on an XF24 cell culture plate (100777-004) at a density of 5 × 10^5^ cells/well with a medium (103575-100) containing 250 μl of Dulbecco’s modified Eagle medium with 5 mM d-glucose and 2 mM glutamine at pH 7.4. After pretreatment, the OCR and ECAR were measured using Seahorse XFe24 Analyzer (Agilent Technologies, Santa Clara, CA, USA).

### Transmission electron microscopy

The scapular BAT was quickly removed and fixed with 2% paraformaldehyde and 2.5% glutaraldehyde in 0.09 M cacodylate buffer (pH 7.2, containing 5% sucrose and 0.025% CaCl_2_). After fixation, the tissues were washed three times with PBS for 30 min, fixed with 1% osmic acid solution for 1 h, and rinsed. Gradient dehydration with ethanol, gradient infiltration with an embedding agent, heating polymerization, ultrathin sectioning, and staining were then performed prior to observation using a JEOL JEM-1400Plus transmission electron microscope.

### Statistical analysis

The results are expressed as mean ± standard error of the mean. A two-tailed Student’s *t*-test was used for comparison between two groups, and one-way analysis of variance and Tukey’s test were used for comparisons among multiple groups with GraphPad Prism 5.0 (GraphPad Software, San Diego, CA, USA). Statistical significance was set at *P* < 0.05.

## Supplementary information

Supplementary data legend.

Supplementary Table 1.

Supplementary Table 2.

Supplementary Figure 1.

Supplementary Figure 2.

Supplementary Figure 3.

Supplementary Figure 4.

Supplementary Figure 5.

Supplementary Figure 6.

Supplementary Figure 7.

Supplementary Figure 8.

Supplementary Figure 9.

Supplementary Figure 10.

Supplementary Figure 11.

Supplementary Figure 12.

## Data Availability

The datasets generated in this study are available from the corresponding author upon request. RNA sequencing data was uploaded to NCBI SRA with accession number PRJNA752947.
