# Author Correction: Trib1 deficiency causes brown adipose respiratory chain depletion and mitochondrial disorder

**DOI:** 10.1038/s41419-024-07066-x

**Published:** 2024-10-14

**Authors:** Xuelian Zhang, Bin Zhang, Chenyang Zhang, Guibo Sun, Xiaobo Sun

**Affiliations:** 1 Institute of Medicinal Plant Development, Peking Union Medical College and Chinese Academy of Medical Sciences, Beijing, 100193 China; 2 Key Laboratory of Bioactive Substances and Resources Utilization of Chinese Herbal Medicine, Ministry of Education, Beijing, 100193 China; 3 Beijing Key Laboratory of Innovative Drug Discovery of Traditional Chinese Medicine (Natural Medicine) and Translational Medicine, Beijing, 100193 China; 4 Key Laboratory of Efficacy Evaluation of Chinese Medicine Against Glyeolipid Metabolism Disorder Disease, State Administration of Traditional Chinese Medicine, Beijing, 100193 China

Correction to: *Cell Death & Disease* 10.1038/s41419-021-04389-x, published online 22 November 2021

Upon reviewing our published work, we realized that we mistakenly used incorrect picture in Fig. 4e (oxidative phosphorylation). To rectify this error, we have prepared the correct version of this figure. The incorrect use of the image does not affect our conclusion.
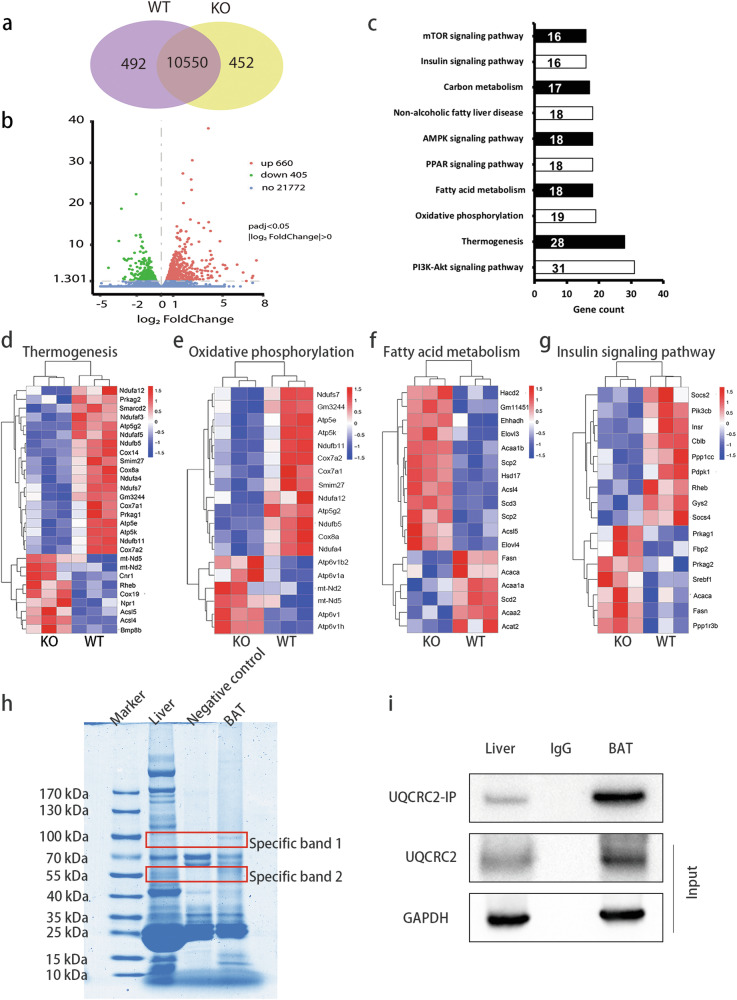

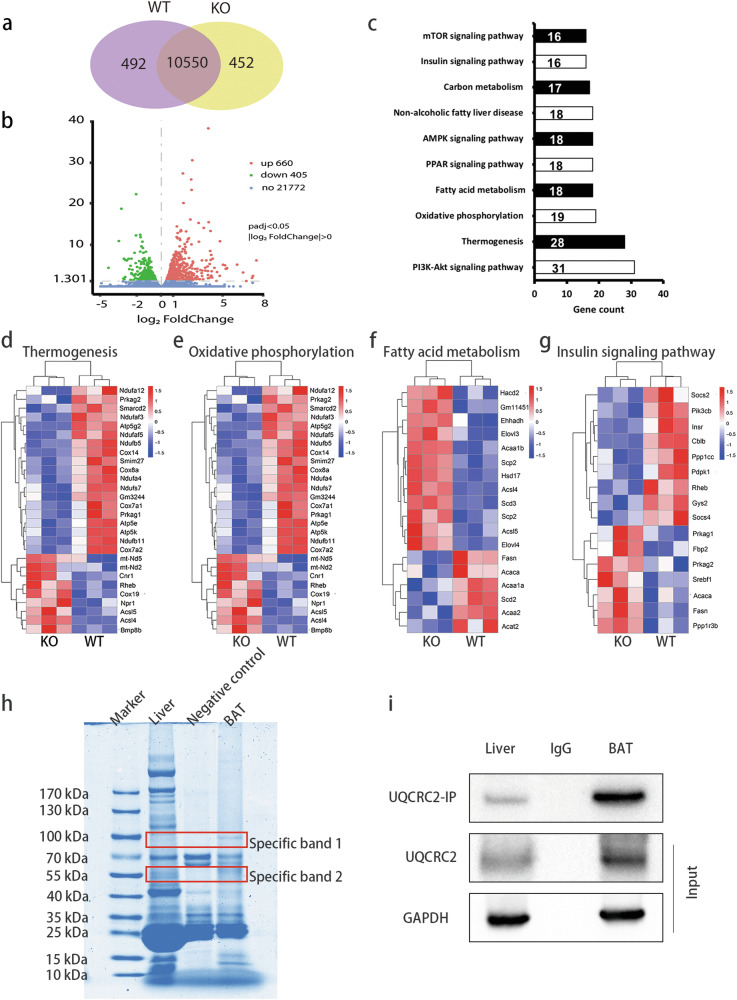


The original article has been corrected.

