# Supplementary material for: Trib1 deficiency causes brown adipose respiratory chain depletion and mitochondrial disorder
Source: Cell Death Dis. 2021 Nov 22;12(12):1098. doi: 10.1038/s41419-021-04389-x (PMC8608845; doi:10.1038/s41419-021-04389-x)
Supplement: Supplementary file 1 — Supplementary data legend. [file 41419_2021_4389_MOESM1_ESM.docx]

**Supplementary Table legend**

**Supplementary Table** **1** Primers utilized for RT-qPCR analysis.

**Supplementary Table** **2** The thermogenesis gene name of heat map from 3T3-L1 cells control group and *Trib1* overexpressing group.

**Supplementary Figure legends**

**Supplementary Figure 1 Expression of *Trib1* in BAT of ob / ob diabetic obese mice and control mice.** In the bar figure, each data represents mean ± SEM (n = 6).

**Supplementary Figure 2 Construction strategy and identification of *Trib1* mice.**

**Supplementary Figure 3** **Representative images of UCP1 immunohistochemistry in adipose tissue of CL316243 treated *Trib1* knockout mice.**

**Supplementary Figure 4** ***Trib1* knockout induced liver injury in mice.** (A-C) Serum AST, ALT, and ALP levels. In the bar figure, each data represents mean ± SEM (n = 4). *P < 0.05, **P < 0.01 over WT mice.

**Supplementary Figure 5** **CL316243 treatment induces hyperlipidemia in *Trib1* knockout mice. (A-C) Serum LDL, TC, and TG levels.** In the bar figure, each data represents mean ± SEM (n = 4). *P < 0.05 over WT mice treated with CL316243.

**Supplementary Figure 6** **Gene Ontology analysis of down regulated genes in brown adipose tissue of *Trib1* knockout mice.**

**Supplementary Figure 7** **Over expression efficiency.** (A) mRNA level of *Trib1* in 3T3-L1 cells. (B) Protein level of *Trib1* in 3T3-L1 cells. In the bar figure, each data represents mean ± SEM (n = 6). *P < 0.05, **P < 0.01 over control group.

**Supplementary Figure 8** **siRNA targeting *Trib1* was transfected into 3T3-L1 adipocytes and stained with oil red.** (A) Red area statistics of 3T3-L1 cells overexpression of *Trib1*. (B, C) Lipid drop oil red staining and red area statistics of 3T3-L1 cells transfected with siRNA targeting *Trib1*. Scale bar: 200 μm. In the bar figure, each data represents mean ± SEM (n = 8). *P < 0.05, **P < 0.01 over control group.

**Supplementary Figure 9 Mitochondrial number was analyzed by fluorescent microscopy using Mitotracker Red.**

**Supplementary Figure 10** **Body weight of wild-type and *Trib1*-haploinsufficient mice fed with high-fat diet.**

**Supplementary Figure 11** **Low survival rate of *Trib1* knockout mice.**

**Supplementary Figure 12** ***Trib1* deficient mice induce increased proinflammatory cytokine gene expression.** (A-C) Serum CRP, TNF-α, and IL-1β levels. In the bar figure, each data represents mean ± SEM (n = 4). *P < 0.05, **P < 0.01, ***P < 0.001 over WT mice.
