# Supplementary material for: Trib1 deficiency causes brown adipose respiratory chain depletion and mitochondrial disorder
Source: Cell Death Dis. 2021 Nov 22;12(12):1098. doi: 10.1038/s41419-021-04389-x (PMC8608845; doi:10.1038/s41419-021-04389-x)
Supplement: Supplementary file 2 — Supplementary Table 1. [file 41419_2021_4389_MOESM2_ESM.docx]

**Supplementary Table 1**

| Gene | Forward Primer | Reverse Primer |
| --- | --- | --- |
| Trib1 | AGAACCCAGCTTAGACTGGAA | AAAAGCGTATAGAGCATCACCC |
| Ucp1 | AGGCTTCCAGTACCATTAGGT | CTGAGTGAGGCAAAGCTGATTT |
| Prdm16 | CCAAGGCAAGGGCGAAGAA | AGTCTGGTGGGATTGGAATGT |
| Cidea | TGACATTCATGGGATTGCAGAC | GGCCAGTTGTGATGACTAAGAC |
| Dio2 | AATTATGCCTCGGAGAAGACCG | GGCAGTTGCCTAGTGAAAGGT |
| Cox7a | GCTCTGGTCCGGTCTTTTAGC | GTACTGGGAGGTCATTGTCGG |
| Ppar-g | TCGCTGATGCACTGCCTATG | GAGAGGTCCACAGAGCTGATT |
| Adipoq | TGTTCCTCTTAATCCTGCCCA | CCAACCTGCACAAGTTCCCTT |
| Cebp-a | CAAGAACAGCAACGAGTACCG | GTCACTGGTCAACTCCAGCAC |
| Leptin | GAGACCCCTGTGTCGGTTC | CTGCGTGTGTGAAATGTCATTG |
| Ndufa9 | ACTGTGTTTGGGGCTACAGG | GATTGATGACCACGTTGCTG |
| SDHA | ACACAGACCTGGTGGAGACC | GCACAGTCAGCCTCATTCAA |
| Uqcrc2 | ATCAAAAGGGGCAACAACAC | CACTCAGGAAGCCCTCTGAC |
| COX1 | ATTCGAGCAGAATTAGGTCA | CTCCGTTATTAGTGGGACA |
| APT5a | AGGCCTATCCTGGTGATGTG | CTTCATGGTACCTGCCACCT |
| OPA1 | CGACTTTGCCGAGGATAGCTT | CGTTGTGAACACACTGCTCTTG |
| MFN1 | ATGGCAGAAACGGTATCTCCA | CTCGGATGCTATTCGATCAAGTT |
| MFN2 | ACCCCGTTACCACAGAAGAAC | AAAGCCACTTTCATGTGCCTC |
| DNM1L | CAGGAATTGTTACGGTTCCCTAA | CCTGAATTAACTTGTCCCGTGA |
| mtDNA | CGAAAGGACAAGAGAAATAGAG | GAACAAGGTTTTAAGTCTTACGCA |
| TFAM | ATTCCGAAGTGTTTTTCCAGCA | TCTGAAAGTTTTGCATCTGGGT |
| CPT1α | GCTGGATGGCTTTGGT | GCTTGGCGGATGTGGTTC |
| LCAD | GGCACAAAAGAACAGATCGAGAA | TGGCTATGGCACCGATACAC |
| MCAD | GCAGAGAAGAAGGGTGACGAGTAT | TCCCCCGTTGGTTATCCA |
| GAPDH | GAGAGTGTTTCCTCGTCCCGTA | TGAGGTCAATGAAGGGGTCG |
