# Supplementary material for: Trib1 deficiency causes brown adipose respiratory chain depletion and mitochondrial disorder
Source: Cell Death Dis. 2021 Nov 22;12(12):1098. doi: 10.1038/s41419-021-04389-x (PMC8608845; doi:10.1038/s41419-021-04389-x)
Supplement: Supplementary file 3 — Supplementary Table 2. [file 41419_2021_4389_MOESM3_ESM.docx]

**Supplementary Table 2** The thermogenesis gene name of heat map from 3T3-L1 cells control group and Trib1 overexpressing group.

| Gene ID | Gene | C1 FPKM | C2 FPKM | C3 FPKM | OE1 FPKM | OE2 FPKM | OE3 FPKM |
| --- | --- | --- | --- | --- | --- | --- | --- |
| ENSMUSG00000064351 | mt-Co1 | 1700.504 | 1682.717 | 1868.665 | 2925.79 | 2721.997 | 3420.504 |
| ENSMUSG00000064370 | mt-Cytb | 582.8285 | 546.0423 | 538.2312 | 901.0624 | 891.1548 | 1118.955 |
| ENSMUSG00000041697 | mt-Nd1 | 553.0393 | 542.0296 | 611.7601 | 606.5512 | 650.0468 | 668.4827 |
| ENSMUSG00000064341 | mt-Nd4 | 424.921 | 416.4039 | 450.4299 | 1240.41 | 1127.675 | 1530.578 |
| ENSMUSG00000064345 | Scd1 | 404.4047 | 394.5141 | 390.7116 | 765.2601 | 757.7668 | 982.5922 |
| ENSMUSG00000064367 | mt-Nd2 | 297.9795 | 291.4868 | 308.7309 | 538.0649 | 554.8749 | 671.4329 |
| ENSMUSG00000025393 | Fasn | 226.1684 | 227.4687 | 242.134 | 265.7624 | 277.1232 | 293.7131 |
| ENSMUSG00000062825 | Fabp4 | 185.7357 | 193.6541 | 192.6567 | 225.5852 | 219.4 | 230.4798 |
| ENSMUSG00000064363 | Lpl | 199.5069 | 185.0342 | 197.3903 | 341.2833 | 341.3016 | 429.466 |
| ENSMUSG00000064368 | Eef1a1 | 188.6397 | 174.366 | 198.6894 | 407.1495 | 447.0466 | 548.599 |
| ENSMUSG00000036751 | Aldoa | 140.6713 | 140.5266 | 146.6215 | 160.4517 | 177.5738 | 182.3863 |
| ENSMUSG00000029580 | mt-Rnr2 | 122.7986 | 121.7719 | 138.254 | 303.0783 | 294.8071 | 313.3337 |
| ENSMUSG00000014294 | mt-Nd5 | 122.3204 | 119.6655 | 119.9857 | 126.2278 | 141.7381 | 140.3973 |
| ENSMUSG00000026895 | C3 | 92.72103 | 100.5891 | 99.0291 | 106.17 | 111.3676 | 114.3986 |
| ENSMUSG00000020163 | Fth1 | 78.66462 | 82.56881 | 88.877 | 128.8081 | 126.4808 | 137.798 |
| ENSMUSG00000036199 | Fn1 | 74.48027 | 82.05958 | 82.33821 | 95.3842 | 96.94782 | 105.8211 |
| ENSMUSG00000018770 | Atp5b | 81.27788 | 79.55569 | 91.89724 | 103.3059 | 111.4552 | 118.1525 |
| ENSMUSG00000044894 | mt-Rnr1 | 78.5804 | 76.99827 | 86.78573 | 103.5084 | 103.8094 | 109.2269 |
| ENSMUSG00000025509 | Bsg | 77.44777 | 70.86534 | 86.77573 | 87.10038 | 96.43567 | 115.5379 |
| ENSMUSG00000013593 | Eef2 | 73.4845 | 70.51319 | 80.18936 | 86.33256 | 90.72602 | 96.21374 |
| ENSMUSG00000071014 | Col1a1 | 57.41073 | 64.21081 | 70.43595 | 81.32545 | 87.12374 | 90.37842 |
| ENSMUSG00000031231 | Col1a2 | 63.87686 | 63.44079 | 68.72222 | 79.14241 | 84.09991 | 88.54812 |
| ENSMUSG00000028648 | Gpd1 | 61.10899 | 62.61554 | 69.71219 | 74.16906 | 81.80756 | 83.17221 |
| ENSMUSG00000061518 | mt-Co2 | 62.24383 | 62.04863 | 70.20592 | 73.15095 | 82.6302 | 83.67535 |
| ENSMUSG00000032330 | Mdm2 | 55.47226 | 59.03372 | 58.32458 | 62.23166 | 70.59587 | 71.6231 |
| ENSMUSG00000029632 | Pkm | 60.84692 | 58.65658 | 67.33347 | 90.73794 | 90.93662 | 94.23612 |
| ENSMUSG00000000171 | Vim | 58.59323 | 55.95215 | 60.69147 | 73.02602 | 75.47036 | 78.83389 |
| ENSMUSG00000016427 | Ucp1 | 60.32135 | 54.11317 | 72.63738 | 115.7458 | 133.8813 | 126.545 |
| ENSMUSG00000021520 | mt-Atp6 | 50.23238 | 52.3301 | 53.72978 | 53.04207 | 63.95763 | 67.95411 |
| ENSMUSG00000026032 | Ero1l | 53.44389 | 50.27557 | 56.77622 | 59.47176 | 66.01855 | 75.86586 |
| ENSMUSG00000050856 | Ldha | 42.09797 | 43.9229 | 45.12475 | 60.4648 | 75.57806 | 62.49301 |
| ENSMUSG00000000399 | Hsp90ab1 | 41.6348 | 40.53172 | 46.18774 | 54.15117 | 55.76079 | 60.13828 |
| ENSMUSG00000059734 | Psap | 35.12441 | 38.94435 | 40.29961 | 45.69341 | 48.99328 | 45.61517 |
| ENSMUSG00000031565 | Col4a1 | 38.08106 | 36.85014 | 42.14949 | 44.53076 | 46.83976 | 46.45978 |
| ENSMUSG00000035674 | Pgk1 | 33.80152 | 35.92219 | 36.51405 | 42.46727 | 46.36857 | 46.2263 |
| ENSMUSG00000022956 | P4hb | 29.88989 | 31.71888 | 31.73971 | 38.54461 | 40.74324 | 45.73617 |
| ENSMUSG00000017188 | Bgn | 27.43087 | 27.74812 | 27.10111 | 37.93503 | 39.70344 | 39.31427 |
| ENSMUSG00000014313 | Sparc | 27.19253 | 25.73988 | 29.95986 | 34.78499 | 39.60305 | 40.76946 |
| ENSMUSG00000026260 | Col6a3 | 26.63689 | 25.4661 | 26.4695 | 28.40553 | 30.36944 | 32.17701 |
| ENSMUSG00000038690 | Eno1 | 22.07317 | 23.5354 | 24.6573 | 29.88465 | 33.73666 | 35.57257 |
| ENSMUSG00000000088 | B2m | 23.5528 | 23.15202 | 25.47787 | 27.1846 | 28.43355 | 29.35517 |
| ENSMUSG00000058076 | Tkt | 25.06505 | 22.71398 | 25.65863 | 29.21038 | 31.82017 | 34.11583 |
| ENSMUSG00000022820 | Thrsp | 22.70483 | 22.60104 | 24.18623 | 25.4015 | 27.08694 | 28.44813 |
| ENSMUSG00000022890 | Col3a1 | 22.1036 | 21.28949 | 22.22028 | 25.85756 | 26.94415 | 28.74462 |
| ENSMUSG00000022994 | H2-D1 | 20.47159 | 20.88999 | 20.4931 | 23.96021 | 23.25389 | 24.19933 |
| ENSMUSG00000064354 | Col4a2 | 21.65791 | 19.6577 | 30.01783 | 58.55549 | 47.21908 | 53.3716 |
| ENSMUSG00000002379 | Glul | 16.7495 | 19.07317 | 22.19871 | 29.80876 | 27.70242 | 32.96785 |
| ENSMUSG00000027230 | Cox6a1 | 17.57874 | 18.90228 | 19.12485 | 29.68484 | 31.01317 | 29.38383 |
| ENSMUSG00000025204 | Gpi1 | 17.9593 | 18.12241 | 21.3416 | 25.39845 | 27.51286 | 27.51432 |
| ENSMUSG00000018796 | Rplp0 | 20.70424 | 18.08893 | 27.32218 | 59.70768 | 74.85013 | 79.43886 |
| ENSMUSG00000025369 | Pnpla2 | 19.36582 | 18.03889 | 19.74419 | 22.22975 | 21.07613 | 21.53474 |
| ENSMUSG00000037152 | Cox4i1 | 13.88941 | 14.3285 | 15.4986 | 21.21801 | 22.76417 | 23.48941 |
| ENSMUSG00000018932 | mt-Nd6 | 15.65018 | 13.92208 | 15.87549 | 21.81298 | 21.73195 | 24.36614 |
| ENSMUSG00000028466 | Cfd | 14.39207 | 13.89357 | 13.08888 | 17.10256 | 16.59989 | 17.05465 |
| ENSMUSG00000027305 | mt-Co3 | 9.91116 | 10.912 | 11.51381 | 12.37856 | 14.37585 | 14.0331 |
| ENSMUSG00000032187 | Aco2 | 10.46233 | 10.83589 | 10.65266 | 15.11699 | 13.76411 | 14.46084 |
| ENSMUSG00000033938 | Tpt1 | 8.512025 | 9.430446 | 9.334685 | 12.66209 | 12.83481 | 14.17666 |
| ENSMUSG00000025499 | Col6a1 | 9.044246 | 9.260747 | 7.968613 | 10.75217 | 11.0868 | 11.96189 |
| ENSMUSG00000038717 | Pck1 | 8.730312 | 8.649361 | 8.984603 | 11.00636 | 11.3772 | 12.70934 |
| ENSMUSG00000064357 | Atp1a2 | 8.784111 | 8.323574 | 10.21846 | 18.79172 | 15.07886 | 18.7809 |
| ENSMUSG00000025968 | Tpi1 | 8.160981 | 7.808908 | 8.629592 | 10.89785 | 10.98752 | 11.26732 |
| ENSMUSG00000031309 | Ftl1 | 7.52686 | 7.377576 | 7.37409 | 8.717359 | 9.216902 | 8.708903 |
| ENSMUSG00000006057 | Hspa9 | 6.618363 | 7.111743 | 8.079791 | 11.94235 | 12.30308 | 12.68461 |
| ENSMUSG00000027673 | Aplp2 | 6.658871 | 6.696673 | 7.356124 | 7.708907 | 7.695347 | 8.478163 |
| ENSMUSG00000064358 | Rpsa | 7.030856 | 6.179165 | 9.071059 | 12.41563 | 17.30153 | 15.70931 |
| ENSMUSG00000032481 | Gpx3 | 5.394496 | 5.886723 | 5.243155 | 7.661691 | 6.480307 | 6.773998 |
| ENSMUSG00000024900 | Fbln2 | 5.484513 | 5.679084 | 4.952767 | 12.72326 | 10.27839 | 11.33933 |
| ENSMUSG00000020516 | Uqcrq | 6.200187 | 5.492591 | 6.297426 | 7.44658 | 7.913588 | 8.473056 |
| ENSMUSG00000024981 | Rplp1 | 3.747494 | 3.882304 | 4.103345 | 8.870043 | 8.936788 | 8.82986 |
| ENSMUSG00000042148 | Cd36 | 3.547523 | 3.353067 | 4.14202 | 5.712601 | 5.505873 | 6.675729 |
| ENSMUSG00000027671 | Gm13394 | 3.014258 | 3.324389 | 3.729044 | 4.877095 | 4.23155 | 4.69714 |
| ENSMUSG00000007880 | Hspa5 | 3.261286 | 2.963587 | 3.088519 | 4.807255 | 4.171694 | 4.683575 |
| ENSMUSG00000069729 | Acly | 2.816248 | 2.780297 | 2.873694 | 3.571807 | 3.422351 | 3.543948 |
| ENSMUSG00000007783 | Cidea | 2.749167 | 2.757565 | 3.09018 | 4.479505 | 4.091191 | 4.636953 |
| ENSMUSG00000028261 | Acadl | 2.13504 | 2.221248 | 2.439907 | 3.397033 | 3.369581 | 3.148262 |
| ENSMUSG00000028495 | Rps2-ps13 | 470.6184 | 485.0242 | 427.9861 | 372.1588 | 386.4194 | 378.4988 |
| ENSMUSG00000031818 | Mt1 | 284.9245 | 302.8296 | 297.0243 | 247.3729 | 259.9406 | 262.3563 |
| ENSMUSG00000017778 | Sdha | 153.6333 | 161.8348 | 155.1036 | 115.5791 | 133.7665 | 125.7047 |
| ENSMUSG00000040048 | Rack1 | 110.634 | 108.0736 | 113.6368 | 88.8275 | 103.5901 | 101.6935 |
| ENSMUSG00000025781 | Hadhb | 104.7258 | 102.9947 | 102.3408 | 78.87977 | 86.60165 | 86.76151 |
| ENSMUSG00000024248 | Plod1 | 97.4542 | 98.08623 | 85.75713 | 73.58696 | 83.30201 | 74.16323 |
| ENSMUSG00000024038 | Gpx4 | 84.83873 | 87.47117 | 85.69862 | 66.28359 | 77.99333 | 74.43432 |
| ENSMUSG00000021577 | Dbi | 85.99514 | 87.16527 | 91.28769 | 72.2039 | 78.46382 | 78.73503 |
| ENSMUSG00000062683 | Ctsb | 80.64477 | 77.04712 | 74.96343 | 54.95057 | 62.07202 | 56.61728 |
| ENSMUSG00000022450 | Txnip | 69.29888 | 71.18106 | 65.53662 | 37.80108 | 46.53794 | 40.73277 |
| ENSMUSG00000022354 | Hspa8 | 66.55724 | 63.86536 | 69.60922 | 51.84319 | 58.31279 | 57.0028 |
| ENSMUSG00000000440 | Cox8b | 57.87648 | 59.07 | 63.8329 | 47.00079 | 53.8524 | 54.99986 |
| ENSMUSG00000020153 | Rps14 | 38.9759 | 41.66441 | 42.39679 | 35.27798 | 38.24554 | 35.69723 |
| ENSMUSG00000050697 | Lgals1 | 35.73962 | 38.61215 | 35.95158 | 18.00767 | 19.74923 | 18.92749 |
| ENSMUSG00000053470 | Gsn | 22.23763 | 22.46987 | 21.12339 | 12.67681 | 12.98667 | 12.20571 |
| ENSMUSG00000031278 | Rpl4 | 21.30637 | 21.09643 | 20.85417 | 13.30017 | 13.28613 | 12.46246 |
| ENSMUSG00000058881 | Hspg2 | 15.22893 | 15.65778 | 14.3852 | 10.8224 | 10.62005 | 10.03102 |
| ENSMUSG00000038205 | Pgam1 | 14.08629 | 14.66703 | 12.95758 | 6.438152 | 8.141899 | 6.358086 |
| ENSMUSG00000038648 | Itm2b | 13.09879 | 13.98676 | 13.12999 | 8.176342 | 8.750297 | 8.6028 |
| ENSMUSG00000074218 | Acaca | 13.46436 | 12.38143 | 12.00651 | 6.742529 | 10.69927 | 7.850663 |
| ENSMUSG00000005034 | Dgat2 | 8.179713 | 8.76861 | 7.823966 | 7.091974 | 7.344077 | 7.07772 |
| ENSMUSG00000034748 | Lrp1 | 8.400536 | 8.519926 | 8.251073 | 7.134118 | 7.150075 | 5.783003 |
| ENSMUSG00000000902 | Ubb | 6.840475 | 7.060716 | 7.338421 | 5.148164 | 5.512153 | 5.157094 |
| ENSMUSG00000032883 | Cav1 | 5.808117 | 6.376297 | 5.388178 | 2.832016 | 2.778829 | 2.232398 |
| ENSMUSG00000030785 | Acaa2 | 4.651832 | 5.912613 | 6.522442 | 2.989203 | 2.805936 | 2.322039 |
| ENSMUSG00000026812 | Sqstm1 | 5.240611 | 5.531118 | 4.885602 | 3.046049 | 2.817426 | 2.898301 |
| ENSMUSG00000038773 | Acsl1 | 4.566236 | 4.596709 | 3.993042 | 3.576847 | 3.179958 | 3.271118 |
| ENSMUSG00000003644 | Ghitm | 4.148442 | 4.489013 | 4.301213 | 2.870967 | 3.096007 | 3.211373 |
| ENSMUSG00000005580 | Rpl8 | 4.091934 | 4.001337 | 3.965493 | 2.809464 | 2.696824 | 2.394153 |
| ENSMUSG00000048351 | Acadm | 3.399494 | 3.414506 | 3.038149 | 2.393803 | 2.657495 | 2.427318 |
| ENSMUSG00000021265 | Cs | 3.280952 | 3.110923 | 3.770681 | 2.438847 | 2.316321 | 2.387263 |
| ENSMUSG00000025958 | Npc2 | 2.240346 | 2.438931 | 2.356647 | 2.12707 | 2.080181 | 2.003895 |
| ENSMUSG00000028944 | Ctsd | 2.43111 | 2.369951 | 2.637507 | 1.802566 | 2.055797 | 2.119936 |
| ENSMUSG00000030584 | Cox8a | 2.475946 | 2.200715 | 2.055416 | 1.613576 | 1.464841 | 1.597784 |
| ENSMUSG00000029167 | Rps3 | 1.444335 | 1.529793 | 1.670461 | 1.224723 | 1.147264 | 1.106632 |
| ENSMUSG00000064351 | mt-Co1 | 1700.504 | 1682.717 | 1868.665 | 2925.79 | 2721.997 | 3420.504 |
| ENSMUSG00000064370 | mt-Cytb | 582.8285 | 546.0423 | 538.2312 | 901.0624 | 891.1548 | 1118.955 |
| ENSMUSG00000041697 | mt-Nd1 | 553.0393 | 542.0296 | 611.7601 | 606.5512 | 650.0468 | 668.4827 |
| ENSMUSG00000064341 | mt-Nd4 | 424.921 | 416.4039 | 450.4299 | 1240.41 | 1127.675 | 1530.578 |
