# Supplementary figures and images for: Trib1 deficiency causes brown adipose respiratory chain depletion and mitochondrial disorder
Source: Cell Death Dis. 2021 Nov 22;12(12):1098. doi: 10.1038/s41419-021-04389-x (PMC8608845; doi:10.1038/s41419-021-04389-x)

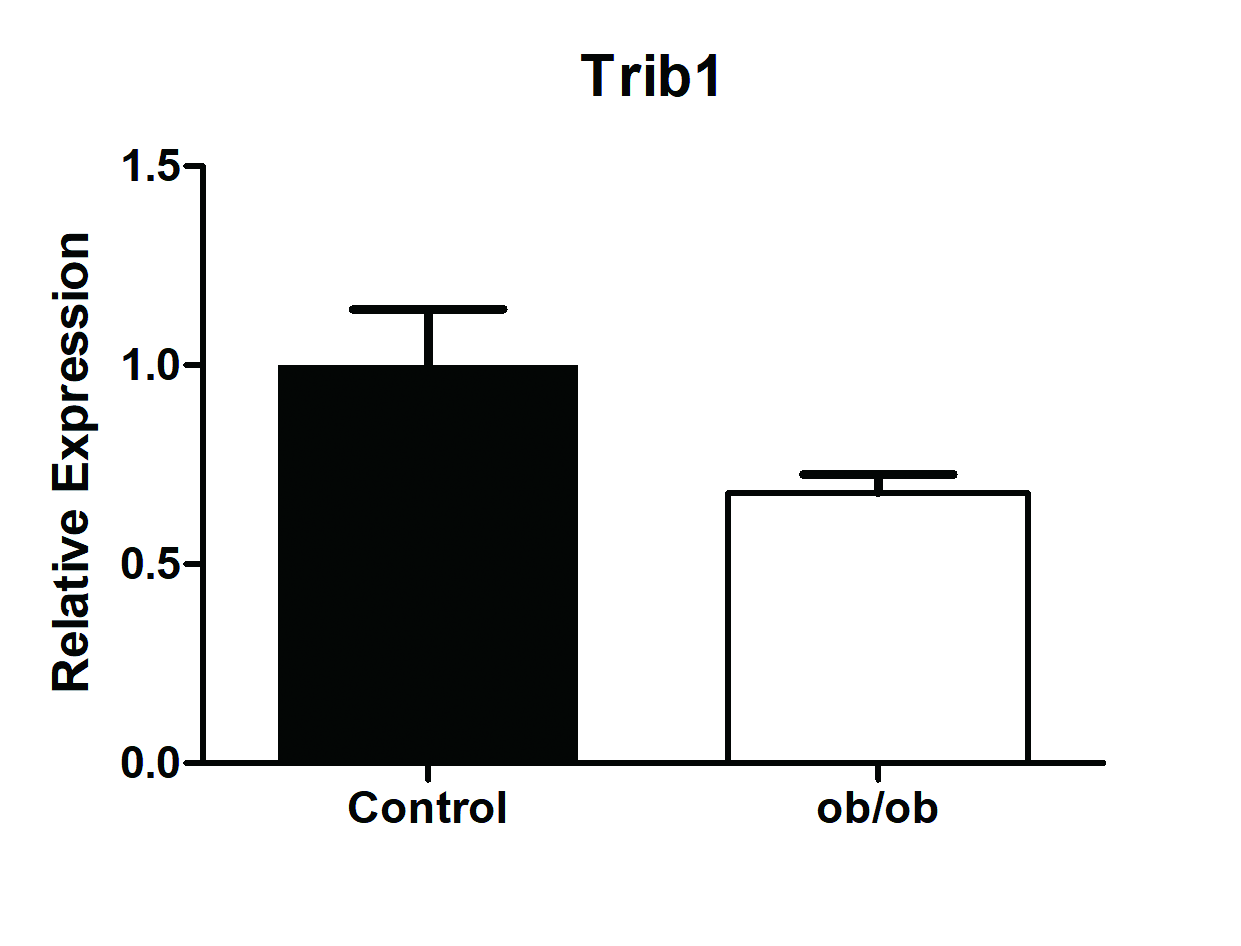

Supplement: Supplementary file 4 — Supplementary Figure 1. [file 41419_2021_4389_MOESM4_ESM.tif]

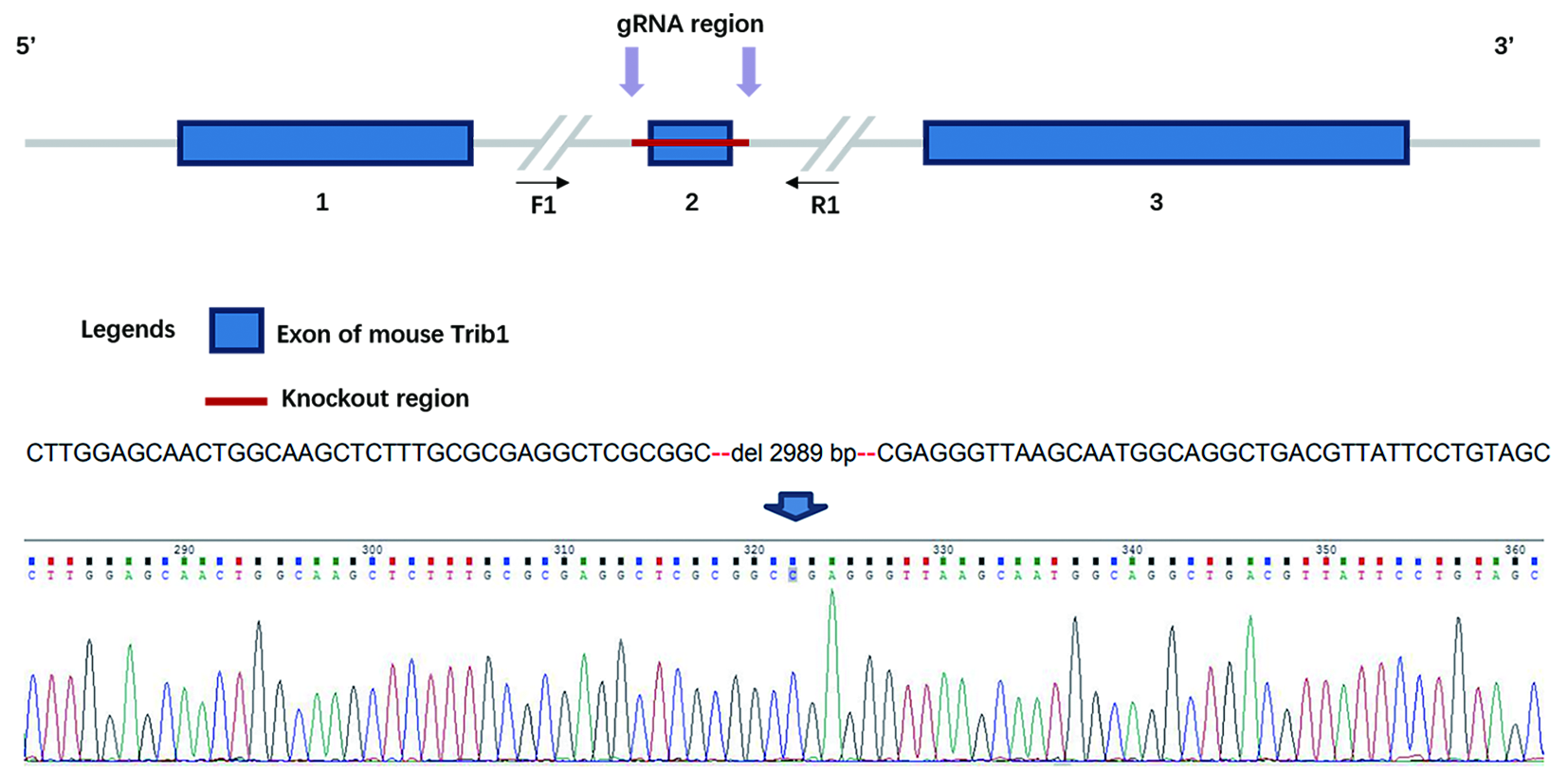

Supplement: Supplementary file 5 — Supplementary Figure 2. [file 41419_2021_4389_MOESM5_ESM.tif]

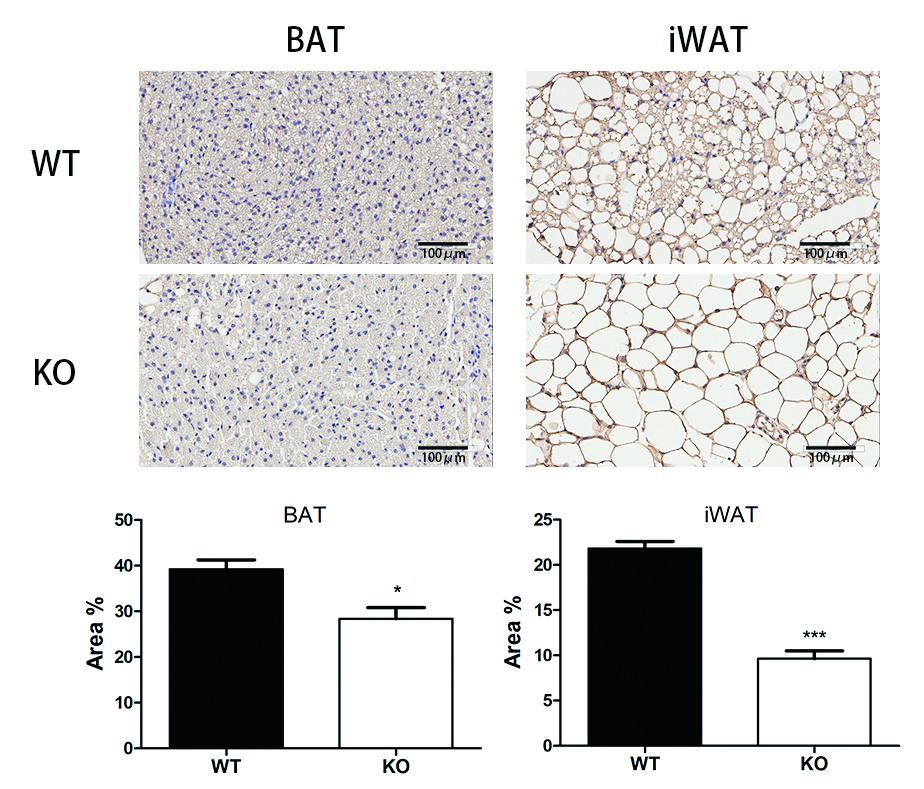

Supplement: Supplementary file 6 — Supplementary Figure 3. [file 41419_2021_4389_MOESM6_ESM.tif]

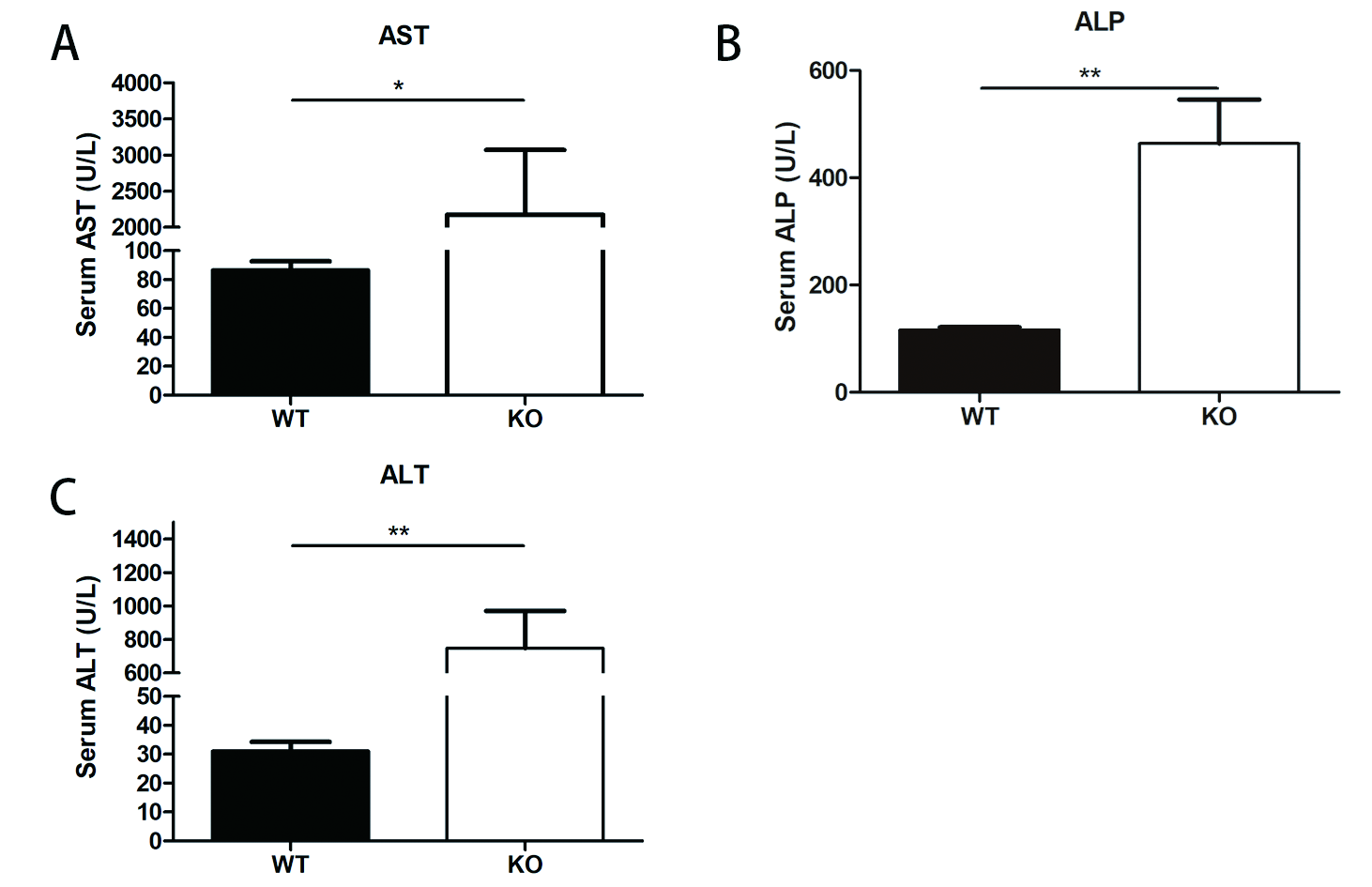

Supplement: Supplementary file 7 — Supplementary Figure 4. [file 41419_2021_4389_MOESM7_ESM.tif]

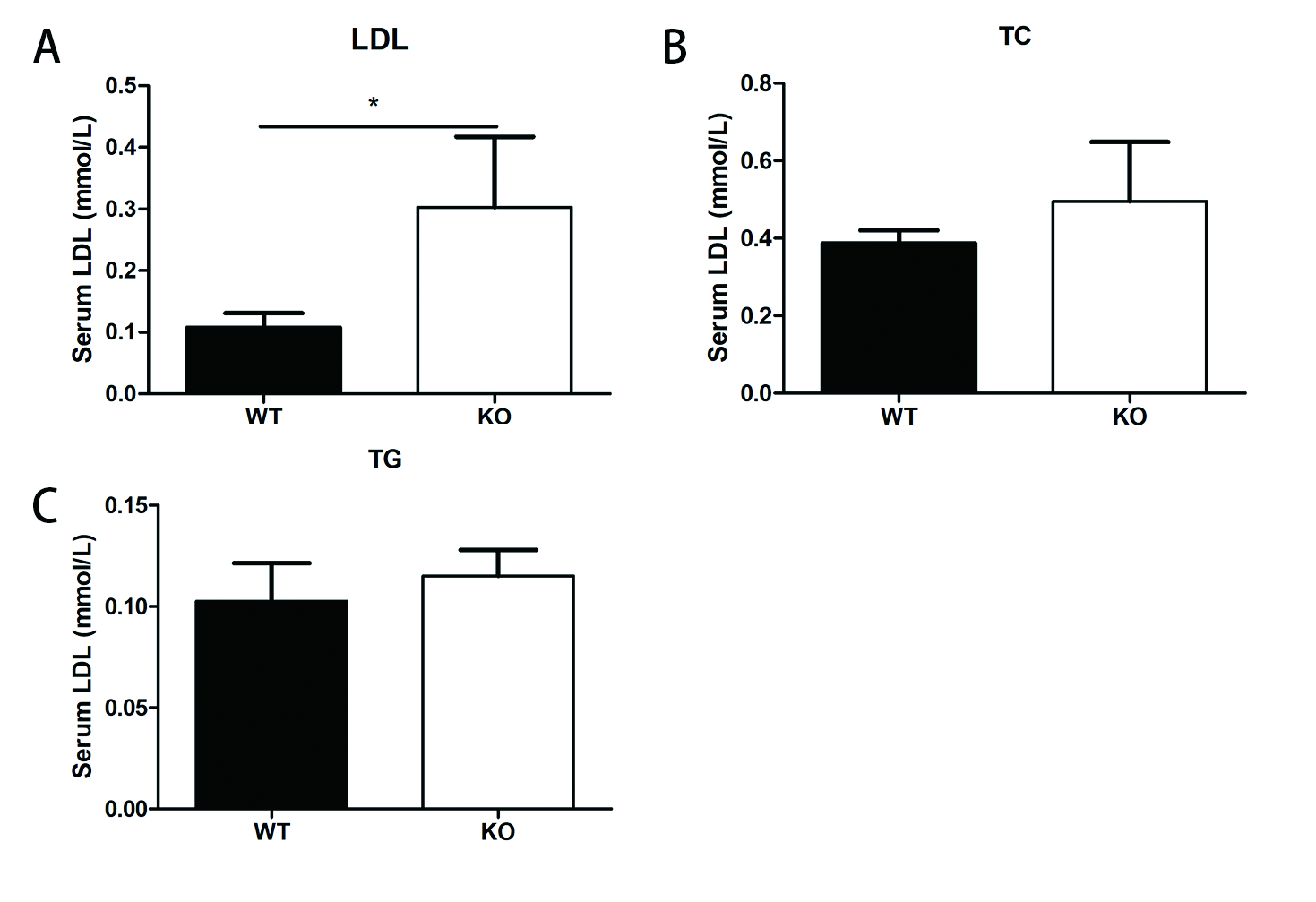

Supplement: Supplementary file 8 — Supplementary Figure 5. [file 41419_2021_4389_MOESM8_ESM.tif]

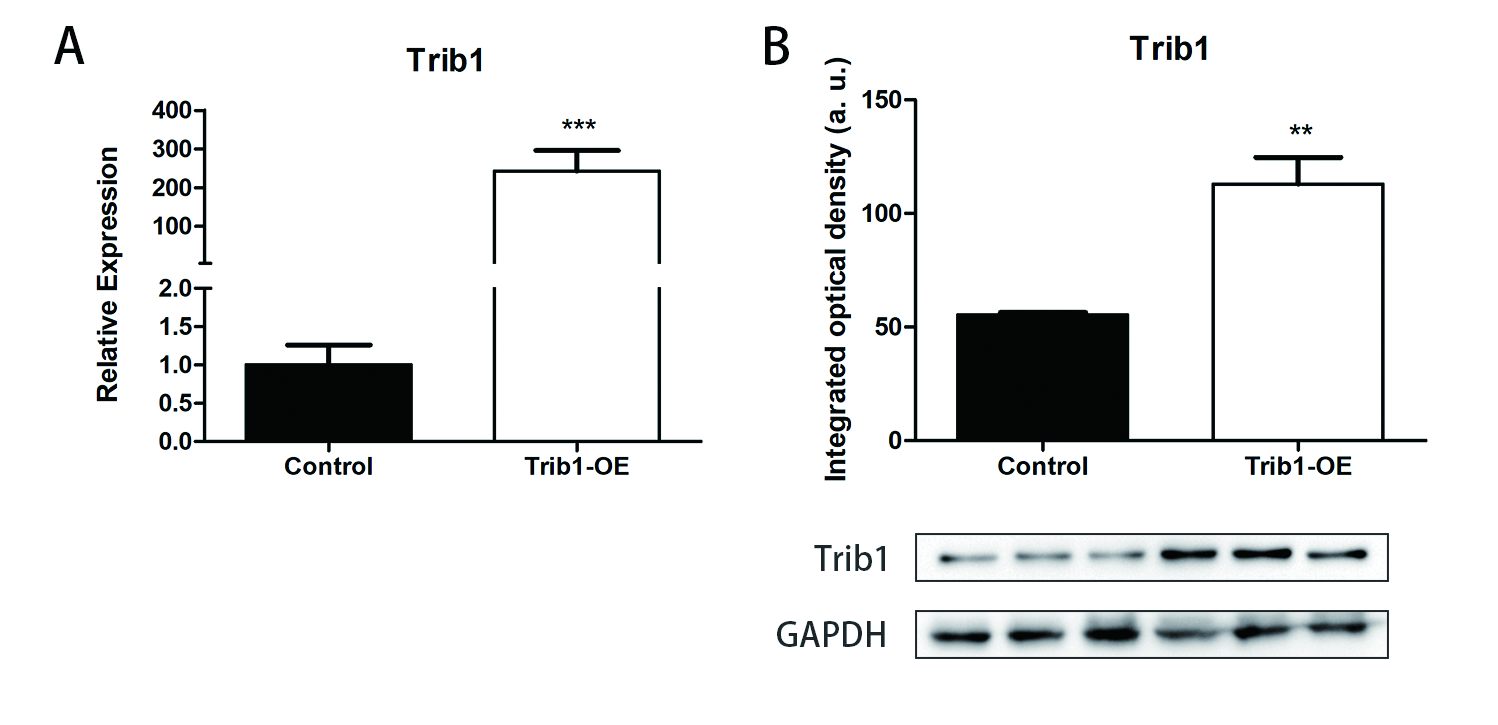

Supplement: Supplementary file 10 — Supplementary Figure 7. [file 41419_2021_4389_MOESM10_ESM.tif]

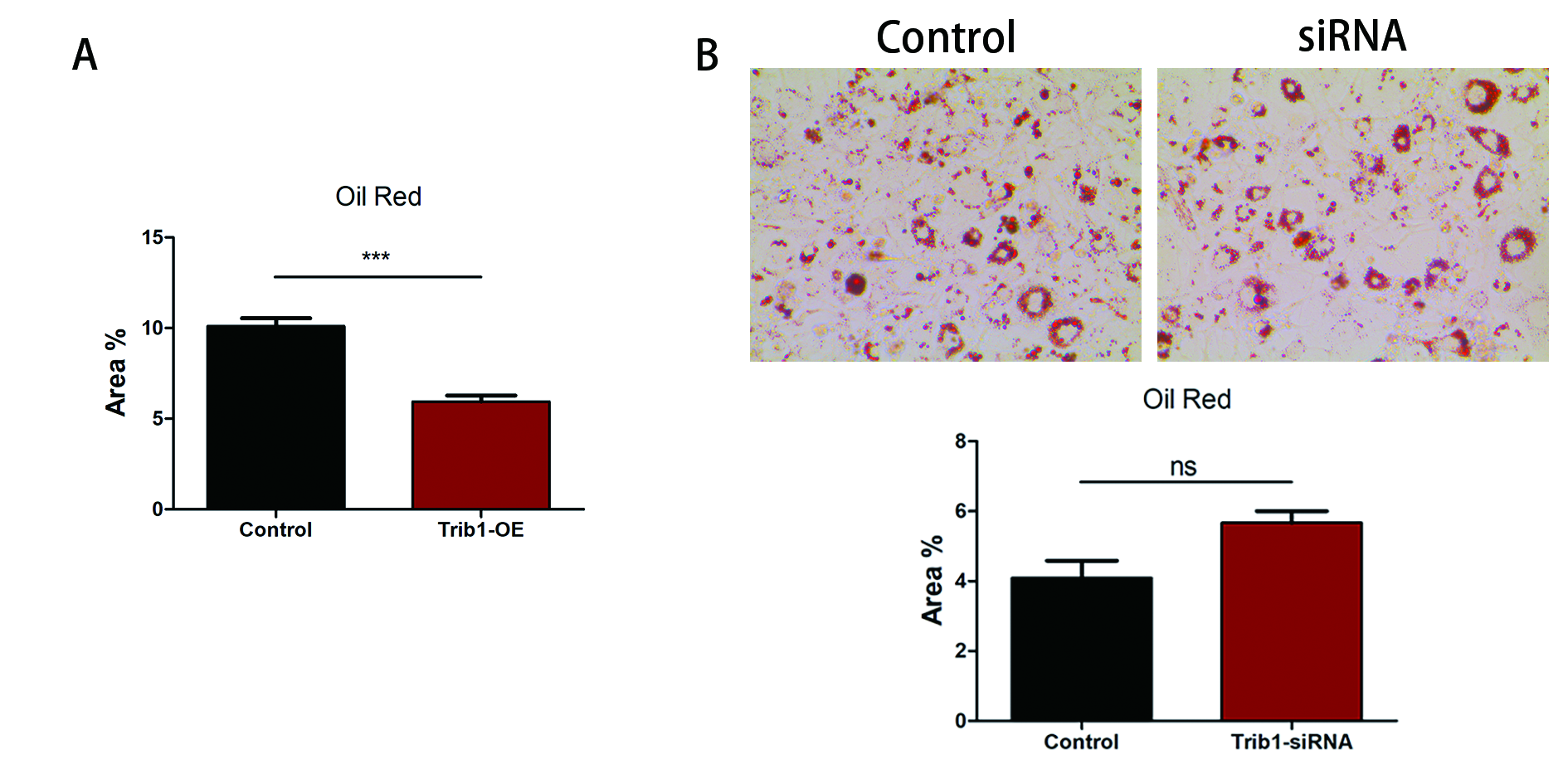

Supplement: Supplementary file 11 — Supplementary Figure 8. [file 41419_2021_4389_MOESM11_ESM.tif]

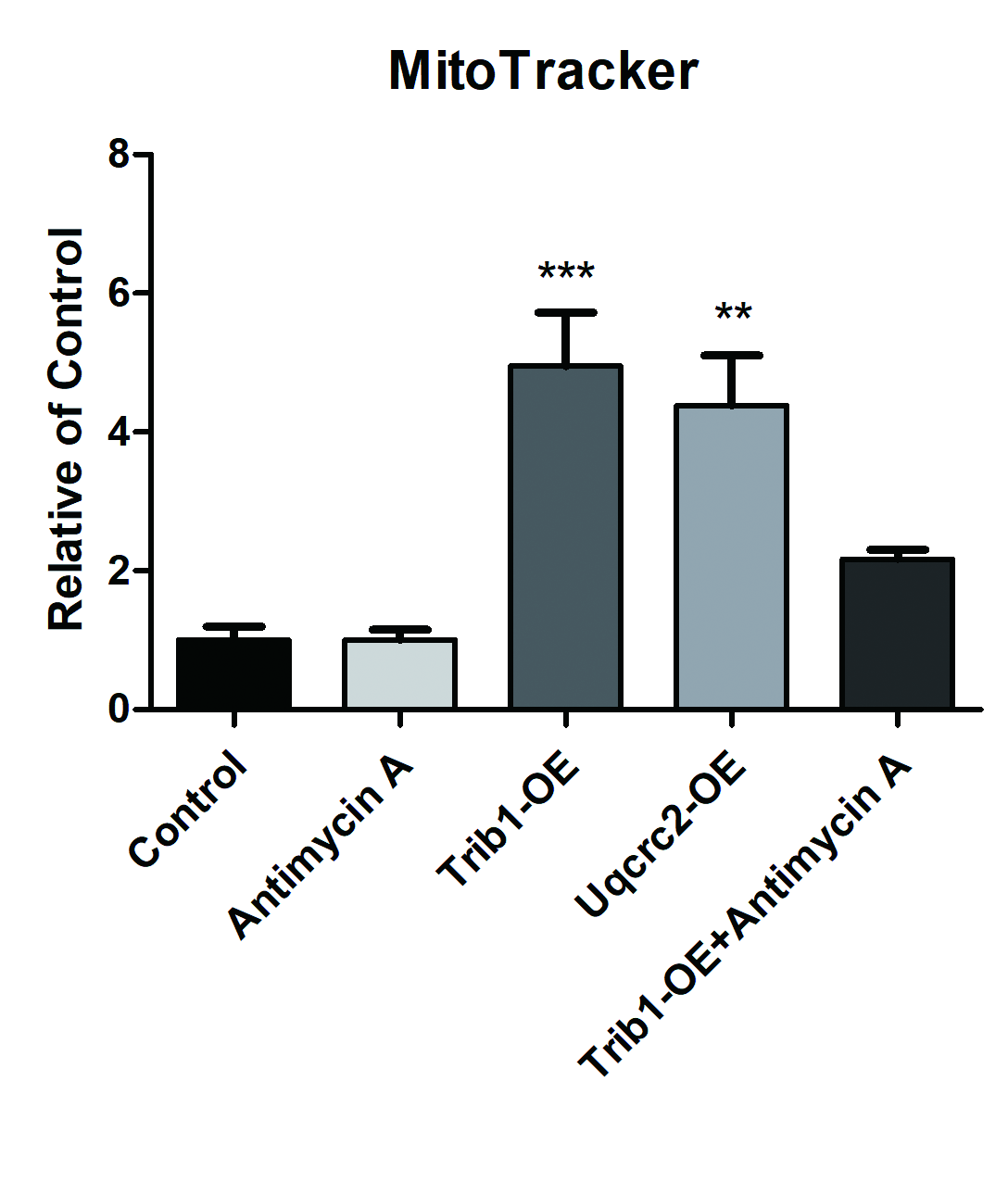

Supplement: Supplementary file 12 — Supplementary Figure 9. [file 41419_2021_4389_MOESM12_ESM.tif]

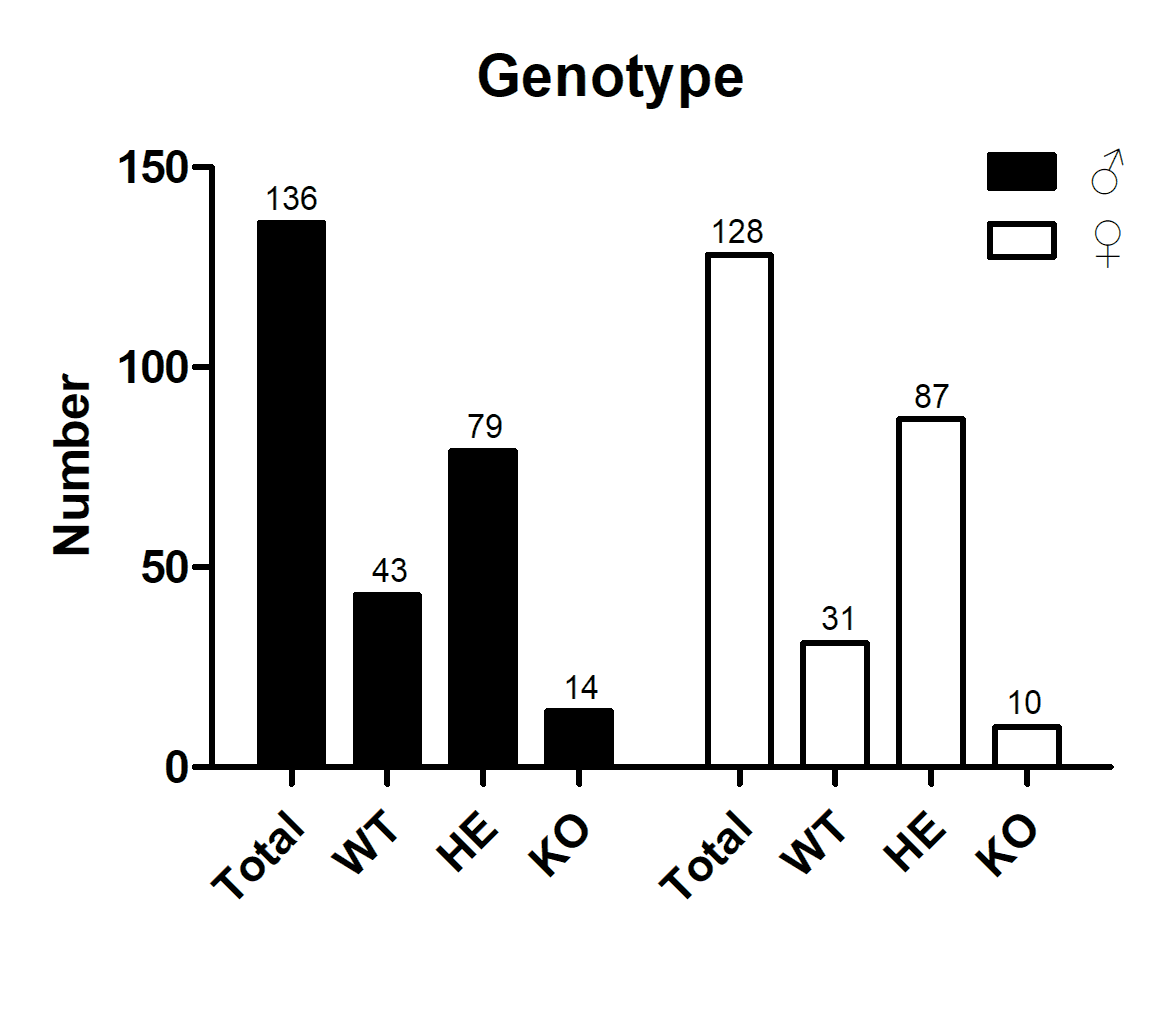

Supplement: Supplementary file 13 — Supplementary Figure 10. [file 41419_2021_4389_MOESM13_ESM.tif]

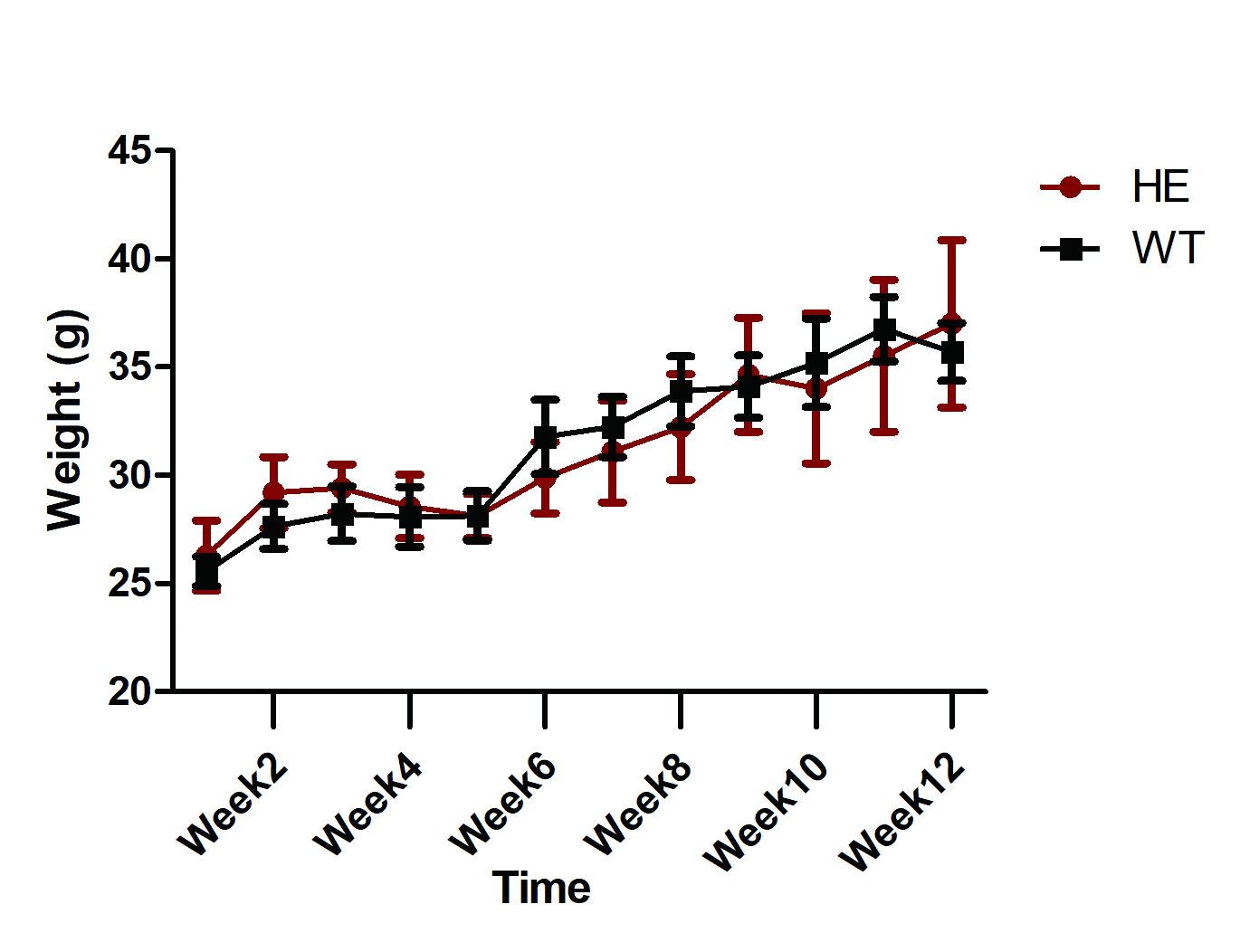

Supplement: Supplementary file 14 — Supplementary Figure 11. [file 41419_2021_4389_MOESM14_ESM.tif]

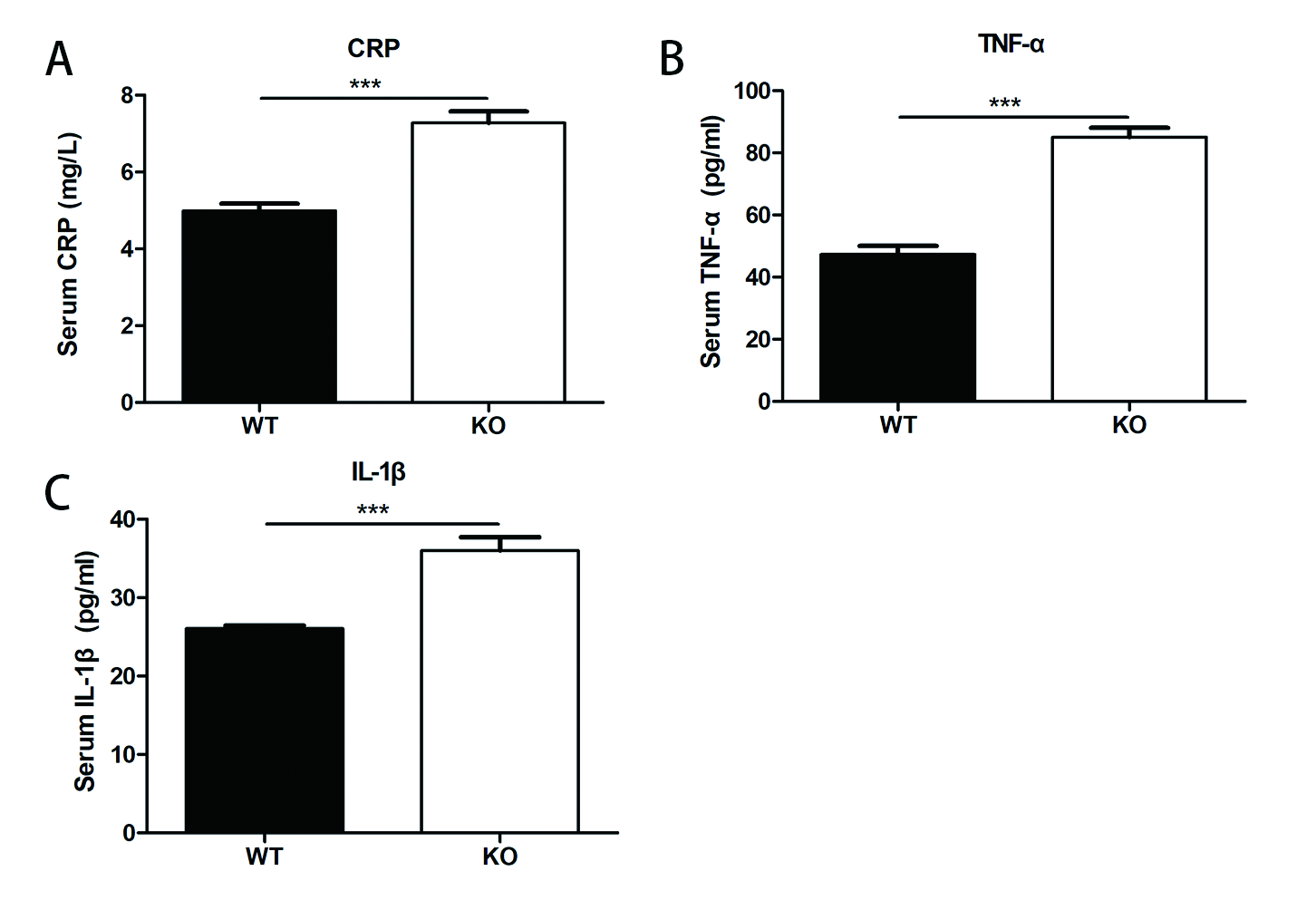

Supplement: Supplementary file 15 — Supplementary Figure 12. [file 41419_2021_4389_MOESM15_ESM.tif]
